# Targeted 2‐Deoxy‐*D*‐Ribose Delivery by Biomimetic Nanoplatform Activates EGFR for Accelerated Heart Valve Endothelialization

**DOI:** 10.1002/advs.202514170

**Published:** 2025-11-12

**Authors:** Xiang Qiu, Gaofeng Li, Wenyi Wan, Jinsheng Li, Ge Yan, Shijie Wang, Xiuqi Hu, Zongqi Han, Yazheng Shan, Ying Zhou, Nianguo Dong, Weihua Qiao

**Affiliations:** ^1^ Department of Cardiovascular Surgery Union Hospital Tongji Medical College Huazhong University of Science and Technology 1277 Jiefang Avenue Wuhan 430022 China; ^2^ Department of Cardiovascular Surgery The Central Hospital of Wuhan Tongji Medical College Huazhong University of Science and Technology Wuhan Hubei 430014 China

**Keywords:** 2‐deoxy*‐D*‐ribose, antibody‐engineered drug delivery, endothelialization, erythrocyte membrane‐camouflaged nanoparticles, tissue‐engineered heart valves

## Abstract

Tissue‐engineered heart valves face clinical translation challenges due to delayed endothelialization. To overcome this, a biomimetic erythrocyte membrane‐camouflaged nanoplatform is engineered to synergistically orchestrate endothelial cell (EC) homing through three mechanisms. The red blood cell membrane coating evades immune clearance and enhances hemocompatibility, while surface‐conjugated CD144 antibodies enable high‐affinity targeting of vascular endothelial cadherin receptors for selective EC adhesion. The poly(lactic‐co‐glycolic acid) core provides sustained release of 2‐deoxy‐D‐ribose, which activates EGFR–MAPK signaling to drive cytoskeletal reorganization and potentiate EC migration/proliferation. In vitro studies demonstrate significantly enhanced EC adhesion strength, directional migration, and proliferative activity. Transcriptomic analysis reveals attenuated TNF‐α/NFκB pathways and upregulated extracellular matrix‐assembly genes. In a rat abdominal aorta model, the platform accelerates formation of a confluent endothelial monolayer within 14 days, with physiological collagen remodeling and minimal thrombus formation. Proteomic profiling confirms downregulated PI3K–Akt‐driven inflammation and neutrophil extracellular trap formation. This multifunctional nanoplatform uniquely bridges antibody‐mediated EC recruitment with 2‐deoxy‐D‐ribose‐induced regenerative signaling, establishing a transformative paradigm for next‐generation tissue‐engineered heart valves with enhanced durability.

## Graphical abstract

This study develops a biomimetic nanoplatform using erythrocyte membrane‐camouflaged, CD144 antibody‐functionalized nanoparticles to deliver 2‐deoxy‐*D‐*ribose (2dDR). This system promotes heart valve endothelialization by enhancing endothelial cell migration and proliferation via EGFR activation, while improving hemocompatibility. In vivo results show accelerated endothelial coverage and reduced thrombosis, offering a promising strategy for next‐generation tissue‐engineered heart valves.

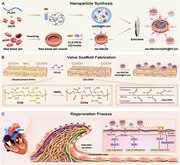

## 1 Introduction

Valvular heart disease represents a major global health burden and arises from diverse etiologies, including rheumatic, degenerative, congenital, infective, and calcific causes, as well as complications such as paravalvular leakage. In patients with symptomatic severe valve dysfunction, the annual mortality rate can reach up to 25% if left untreated.^[^ ^1^ ^]^ Valve replacement remains the only effective solution. Current valve substitutes, including mechanical and bioprosthetic valves, present significant limitations. Mechanical valves offer excellent durability but require lifelong anticoagulation, which increases the risk of hemorrhagic complications.^[^ ^2^, ^3^ ^]^ Bioprosthetic valves, while avoiding the need for anticoagulation, are susceptible to structural valve deterioration, parti in younger patients, which limits their long‐term efficacy.^[^ ^4^, ^5^ ^]^ In this context, tissue‐engineered heart valves (TEHVs) have emerged as a promising alternative, aiming to replicate the native valve architecture by promoting host‐cell repopulation within the scaffold and supporting physiological remodeling to achieve durable function.^[^ ^6^ ^]^

Decellularized heart valves (DHVs) have demonstrated promising structural and biomechanical properties for valve replacement, as they preserve the native extracellular matrix (ECM) architecture and mechanical strength of natural valves.^[^ ^7^ ^]^ Preclinical studies in large‐animal models and early clinical trials have shown that these valves can support near‐physiological hemodynamics and reduce early calcification.^[^ ^8^ ^]^ However, their clinical translation remains limited, largely due to delayed or incomplete endothelialization, which is a key factor contributing to thrombus formation, chronic inflammation, and eventual valve degeneration.^[^ ^9^, ^10^, ^11^ ^]^ Although decellularization is necessary to reduce immunogenicity, it removes essential bioactive cues that promote the adhesion, migration, and proliferation of endothelial cells (ECs).^[^ ^12^, ^13^, ^14^ ^]^ To address this challenge, numerous strategies have aimed to capture circulating endothelial progenitor cells (EPCs) from the bloodstream and harness their ability to differentiate into mature ECs. Approaches such as surface modification with EPC‐targeting peptides, antibodies (e.g., anti‐CD34), or growth factors have been explored;^[^ ^15^, ^16^, ^17^ ^]^ however, these methods have yielded limited success owing to the low abundance of EPCs in adult circulation and their poor adhesion to bioinert scaffolds.^[^ ^18^ ^]^ As a result, endothelialization remains inefficient and slow, compromising the long‐term functionality of TEHVs.^[^ ^19^, ^20^, ^21^ ^]^ Emerging evidence suggests that peripheral resident ECs in the adjacent vascular endothelium play an important role in surface endothelialization. These mature ECs can migrate toward the implant site when guided by appropriate biochemical and biomechanical cues provided by bioactive scaffolds, ultimately forming a functional endothelial monolayer.^[^ ^22^, ^23^ ^]^ Therefore, developing biomaterials or delivery systems that promote directional endothelial migration represents a promising strategy to accelerate valve endothelialization and improve the durability of TEHVs.

2‐Deoxy‐*D*‐ribose (2dDR) has been identified as a potent angiogenic agent that promotes the proliferation, migration, and tube formation of human aortic ECs.^[^ ^24^, ^25^ ^]^ In vitro assays have shown that 2dDR enhances EC function in a dose‐dependent manner, highlighting its potential to accelerate the endothelialization processes critical for TEHVs.^[^ ^26^, ^27^, ^28^ ^]^ To date, this application has been limited to preliminary studies in wound healing and capillary regeneration,^[^ ^29^ ^]^ with no reports on promoting the endothelialization of cardiovascular implants. The localized delivery of 2dDR offers a promising strategy to provide bioactive cues that direct resident EC migration and adhesion, thereby accelerating the endothelialization of TEHVs.

In addition to these properties, 2dDR (2‐deoxy*‐D*‐ribose) was selected for its superior stability compared with traditional cytokines such as SDF‐1α, MCP‐1, VEGF, and HIF‐α, allowing sustained stimulation of endothelial proliferation and migration under in vitro conditions.^[^ ^30^ ^]^ It also features low synthetic cost and excellent scalability for large‐scale applications.^[^ ^31^ ^]^ Furthermore, 2dDR has been reported to upregulate VEGF expression within endothelial cells, establishing a positive feedback loop that promotes angiogenesis without the need for genetic modification.^[^ ^24^ ^]^ These advantages make 2dDR an ideal small‐molecule bioactive agent for long‐term endothelial activation.

Regarding CD144 (VE‐cadherin), we selected this target due to its strict endothelial specificity and critical role in maintaining vascular integrity and intercellular junctions. CD144 is broadly yet specifically expressed in EPCs, endocardial cells, and circulating endothelial cells, making it a robust and reliable endothelial marker.^[^ ^32^, ^33^ ^]^ Previous studies have shown that CD144‐based sorting can enrich endothelial or endocardial populations from cardiac tissues,^[^ ^34^ ^]^ and Cdh5(CD144)‐CreER tracing confirms stable expression in valve endothelium throughout postnatal and adult stages.^[^ ^35^ ^]^ Therefore, surface modification with CD144 antibodies can improve endothelial capture efficiency, enhance re‐endothelialization, and promote the biological integration of tissue‐engineered heart valve scaffolds.

Despite advances in drug delivery systems for cardiovascular applications, conventional strategies remain limited by off‐target effects, rapid systemic clearance, and insufficient retention at the implantation site.^[^ ^36^, ^37^ ^]^ Although these systems can encapsulate and release therapeutic agents, they are often recognized and rapidly cleared by the mononuclear phagocyte system, leading to limited delivery efficacy.^[^ ^38^ ^]^ To address these challenges, biomimetic nanoplatforms camouflaged with erythrocyte membranes have gained increasing attention. Camouflaging nanoplatforms with erythrocyte membranes, such as red blood cell membranes (RBCMs), has emerged as a promising strategy owing to their innate immunoevasive properties, prolonged circulation, and excellent hemocompatibility.^[^ ^39^, ^40^ ^]^ This approach reduces immune recognition, stabilizes drug delivery, and decreases platelet adhesion and activation, minimizes thrombus formation, and promotes endothelial adhesion and migration.^[^ ^41^, ^42^, ^43^, ^44^ ^]^

**Scheme 1:**
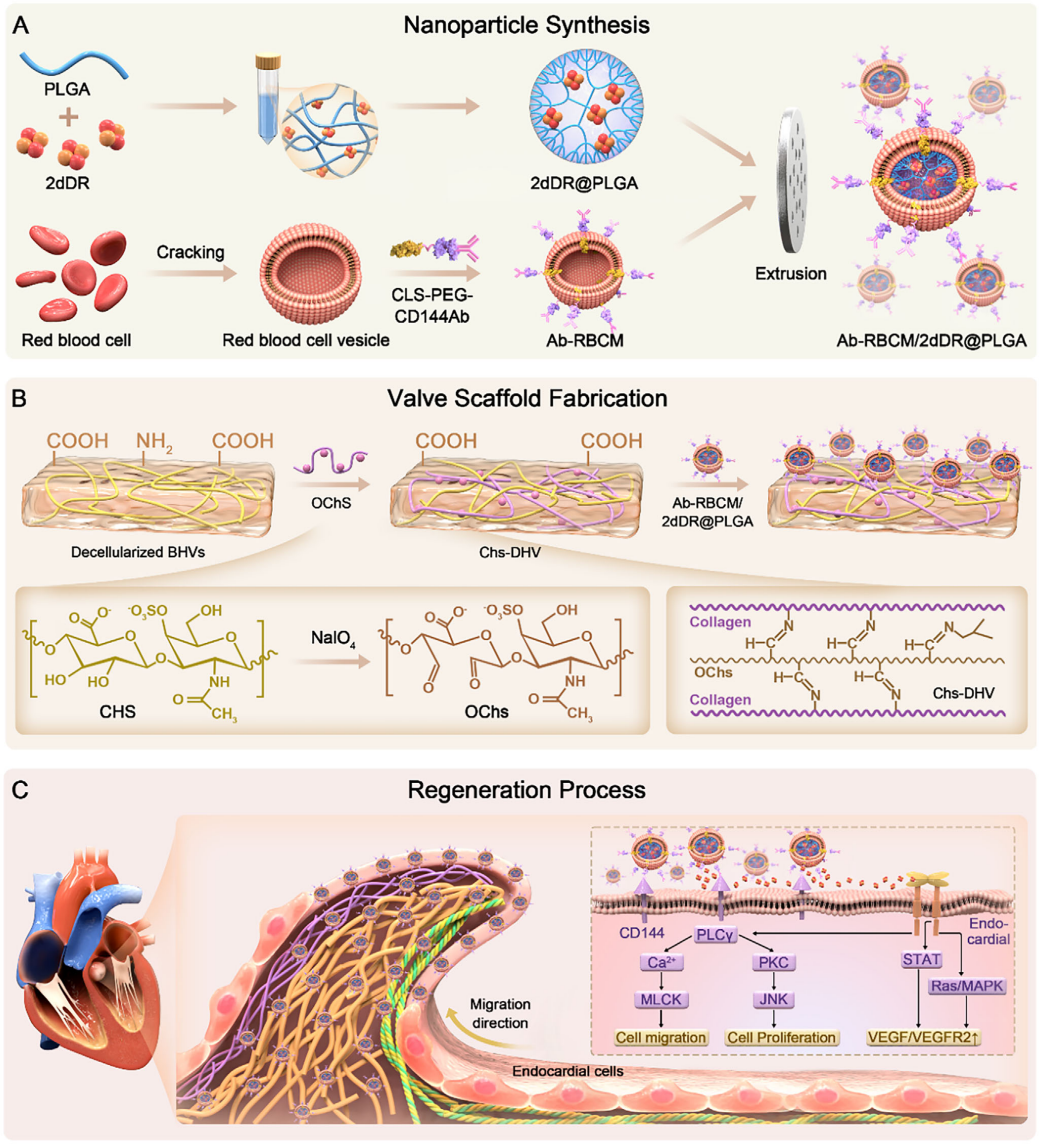
Schematic illustrations of nanoparticle (NP) synthesis and their application in heart valve regeneration. A) Schematic of NP synthesis. B) Schematic of the preparation and NP loading of decellularized heart valve (DHV) scaffolds. C) Schematic illustrating how NP‐loaded scaffolds promote heart valve regeneration.

CD144, also known as vascular endothelial cadherin (VE‐cadherin), is predominantly expressed on the surface of ECs. Monoclonal antibodies targeting CD144 can modulate EC adhesion and junctional permeability,^[^ ^42^, ^45^ ^]^ potentially enhancing the integration of ECs into TEHV scaffolds and improving endothelialization. To further enhance the targeting specificity, the biomimetic nanoplatform was surface‐modified with endothelial‐specific ligands. In this study, CD144 antibodies were used to functionalize nanoparticles (NPs) loaded with 2dDR and coated with RBCMs. This dual strategy combines the stealth and hemocompatibility of erythrocyte membranes with the selective targeting capability of CD144, enabling precise delivery of 2dDR to ECs. This integrated approach achieved rapid and stable endothelial coverage, modulated inflammatory responses, and met multiple key criteria for next‐generation clinical TEHVs (**Scheme** **1**).

## 2 Results

### 2.1 Preparation and Characterization of Ab‐RBCM/2dDR@PLGA NPs

**Figure 1:**
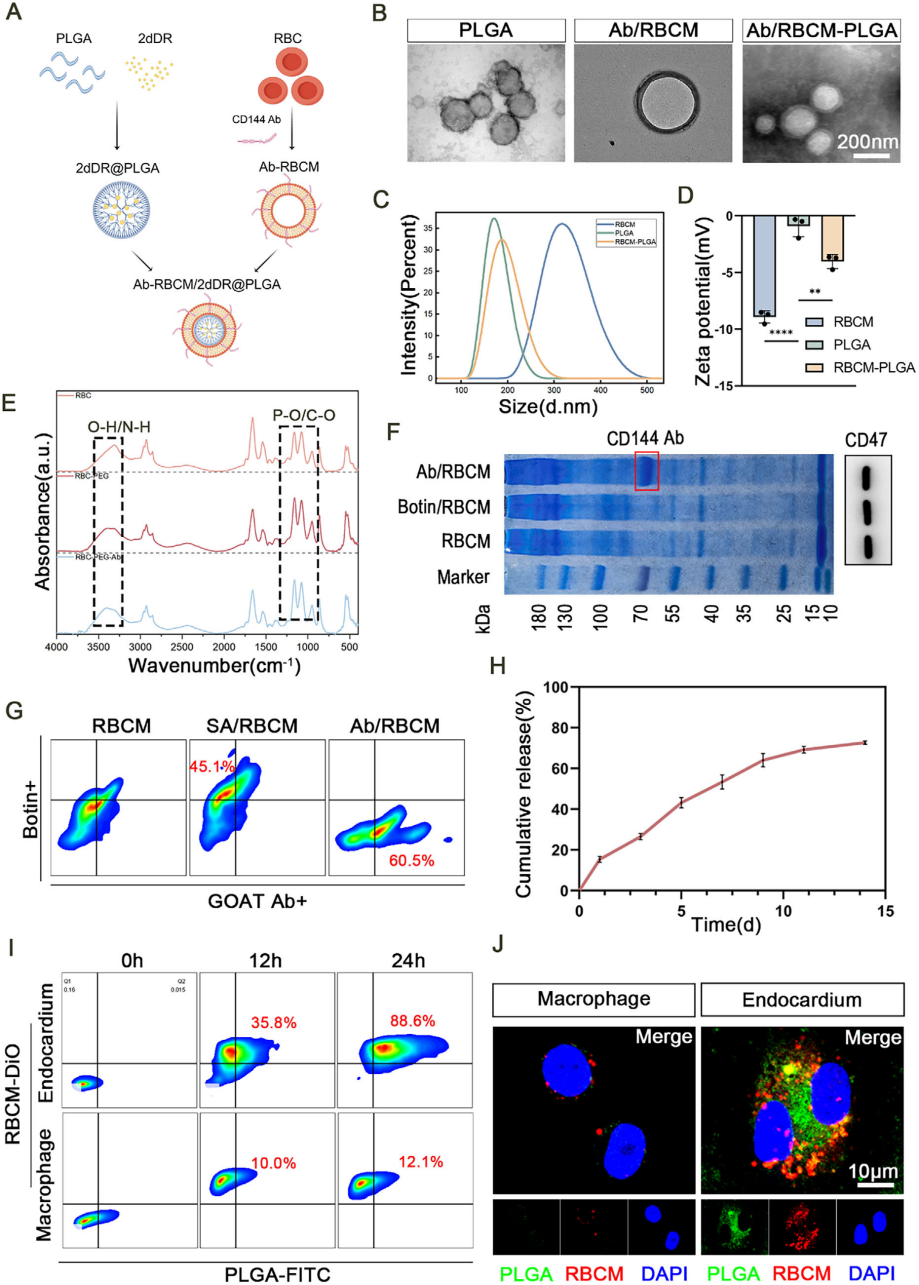
Fabrication and characterization of antibody‐functionalized red blood cell membrane‐camouflaged NPs (Ab‐RBCM)/2‐deoxy*‐D*‐ribose (2dDR)@poly(lactic‐*co‐*glycolic acid) (PLGA). A) Schematic representation of the fabrication process. B) TEM images of NPs at different fabrication stages. (Scale bars: 200 nm). C) Dynamic Light Scattering (DLS) size distribution, D) zeta potential measurements, and E) Fourier‐transform infrared spectra of NPs at different fabrication stages. (*n* = 3). F) Coomassie blue staining (left) and CD47 Western blot analysis (right) of RBCMs during antibody modification. G) Flow cytometric analysis of biotinylated red blood cells with streptavidin–phycoerythrin and Ab‐FITC. H) 2dDR release profile of Ab‐RBCM/2dDR@PLGA. I) Flow cytometric analysis after co‐culture with the endocardium (upper) and macrophages (lower). J) Confocal fluorescence microscopy images after 24 h of co‐culture with the endocardium and macrophages. (Scale bars: 10 µm). Significance levels are denoted as **P < 0.01; ****P < 0.0001.

To construct a biomimetic erythrocyte membrane‐camouflaged nanoplatform, poly(lactic*‐co‐*glycolic acid) (PLGA) NPs, which are approved by the U.S. FDA for drug delivery applications^[^ ^46^ ^]^ were coated with VE‐cadherin antibody‐modified RBCMs via biotin–streptavidin coupling. VE‐cadherin antibodies were linked to the red blood cell (RBC) surface (RBC‐Ab) using a bifunctionalized DNA linker (5′‐Chol‐DNA‐Biotin‐3′).^[^ ^47^ ^]^ The resulting nanoplatform exhibited notable biodegradability and a high drug‐loading capacity.^[^ ^48^, ^49^, ^50^ ^]^ The incorporation of biotinylated VE‐cadherin (CD144) antibodies enabled precise 1:1 molar anchoring onto the RBCM surface. Successful antibody conjugation was confirmed through streptavidin–phycoerythrin and FITC‐labeled antibody binding assays (**Figure** **1**A). Scanning electron microscopy (SEM) revealed the presence of a continuous RBCM layer on the NP surface (Figure 1B). After extrusion, the particle diameter stabilized at ≈200 nm (Figure 1C), and zeta potential measurements showed a slight decrease, indicating increased colloidal stability (Figure 1D). PEGylation further modified the surface, as evidenced by changes in the protein and polysaccharide distributions (Figure 1E). SDS‐PAGE analysis showed preservation of the membrane protein profiles across the RBCM, RBCM‐biotin, and RBCM‐Ab groups. A distinct VE‐cadherin antibody band observed in the RBCM‐Ab sample confirmed successful conjugation without disrupting native proteins. Western blot analysis confirmed the presence of CD47 in both RBCM and RBCM‐coated NPs, indicating the successful retention of this membrane marker following NP functionalization. Since only the RBCM fraction was used, β‐actin and other cytoplasmic housekeeping proteins were not available as internal controls. Therefore, CD47 was presented as a qualitative indicator to validate the coating process, consistent with previous reports on RBCM‐coated nanoplatforms (Figure 1F).

CD47, a “self‐marker” expressed on RBCs, interacts with Signal regulatory protein alpha on macrophages to deliver a “don't eat me” signal that inhibits phagocytosis and promotes immune evasion.^[^ ^51^ ^]^ Flow cytometry analysis revealed that after 12 h of co‐incubation with nanoparticles, the proportion of DiO/FITC double‐positive endothelial cells reached 35.8%, while macrophages exhibited only 10% positivity. At 24 h, the rates increased to 88.6% for endothelial cells and 12.1% for macrophages, respectively, indicating a strong targeting preference toward endothelial cells with minimal uptake by macrophages. Fluorescence imaging corroborated these findings: after 24 h, most nanoparticles (green) co‐localized with the RBCM membrane (red) in endothelial cells (Figure 1I,J), while little signal overlap was observed in macrophages.

The drug‐loading efficiency (DL%) of Ab‐RBCM/2dDR@PLGA was 7.51%, and the encapsulation efficiency (EF%) was 5.43%, confirming the successful encapsulation of 2dDR within the engineered nanosystem, consistent with previous reports on PLGA nanoparticles encapsulating hydrophilic small molecules.^[^ ^52^, ^53^, ^54^ ^]^

### 2.2 Ab‐RBCM/2dDR@PLGA NPs Promote EC Proliferation and Migration via EGFR Activation

**Figure 2:**
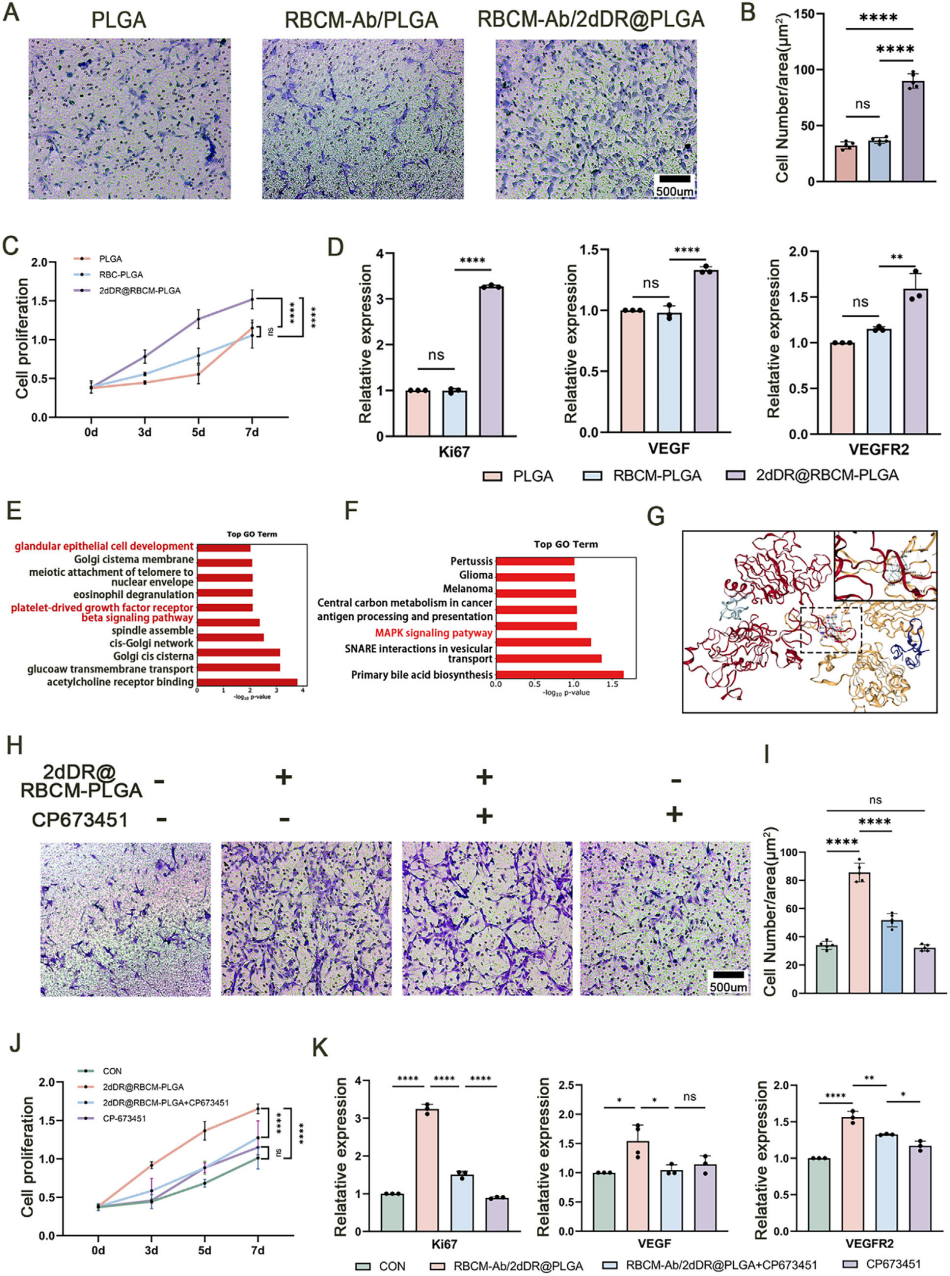
Mechanism of Ab‐RBCM/2dDR@PLGA NPs in accelerating endothelialization. A,B) Transwell assays showing the migration of endocardial cells co‐cultured with NPs. (*n* = 5; Scale bars: 500 µm). C) Endocardial cell proliferation measured by a CCK‐8 assay. (*n* = 3). D) mRNA expression levels of the vascular endothelial growth factor (VEGF), VEGF receptor 2 (VEGFR2), and Ki67 in endocardial cells after co‐culture with NPs. (*n* = 3). E) Gene Ontology (GO) terms and F) Kyoto Encyclopedia of Genes and Genomes (KEGG) enrichment of highly expressed genes in the Ab‐RBCM/2dDR@PLGA group versus the control. G) Binding mode of 2dDR within C1 showing multiple hydrogen bonds with key amino acid residues. H,I) Transwell assay. (*n* = 5; Scale bars: 500 µm). (J) CCK‐8. K) mRNA level after co‐culture with NPs and the epidermal growth factor receptor. (*n* = 3). Significance levels are denoted as ns, non‐significant; *P < 0.05; **P < 0.01; ****P < 0.0001.

A series of in vitro functional assays was performed to determine whether Ab‐RBCM/2dDR@PLGA NPs could enhance EC migration and repair. Transwell migration and wound healing assays demonstrated that 2dDR‐loaded NPs markedly enhanced EC motility compared with those of both the untreated controls and drug‐free NP‐treated groups (**Figures** **2**A,B; S1, Supporting Information). The number of migrating cells was significantly higher in the 2dDR NP group than in either the control or drug‐free NP groups. Consistently, the CCK‐8 assay showed that the cell proliferation rate in the 2dDR NP group was significantly increased after 24 h of culture compared with those in the untreated control and drug‐free NP groups (Figure 2C). Quantitative analysis of absorbance values in the 2dDR‐treated group indicated that the 2dDR‐loaded NPs significantly enhanced the early proliferative activity of ECs. qPCR further revealed that treatment with 2dDR NPs significantly upregulated the expression of proliferation‐ and angiogenesis‐related genes in ECs (Figure 2D). Specifically, the mRNA expression levels of Ki67 (a proliferation marker), vascular endothelial growth factor (VEGF), and VEGF receptor 2 (VEGFR2) were significantly higher in the 2dDR NP group compared with those in the control (untreated) and drug‐free NP groups.

**Figure 3:**
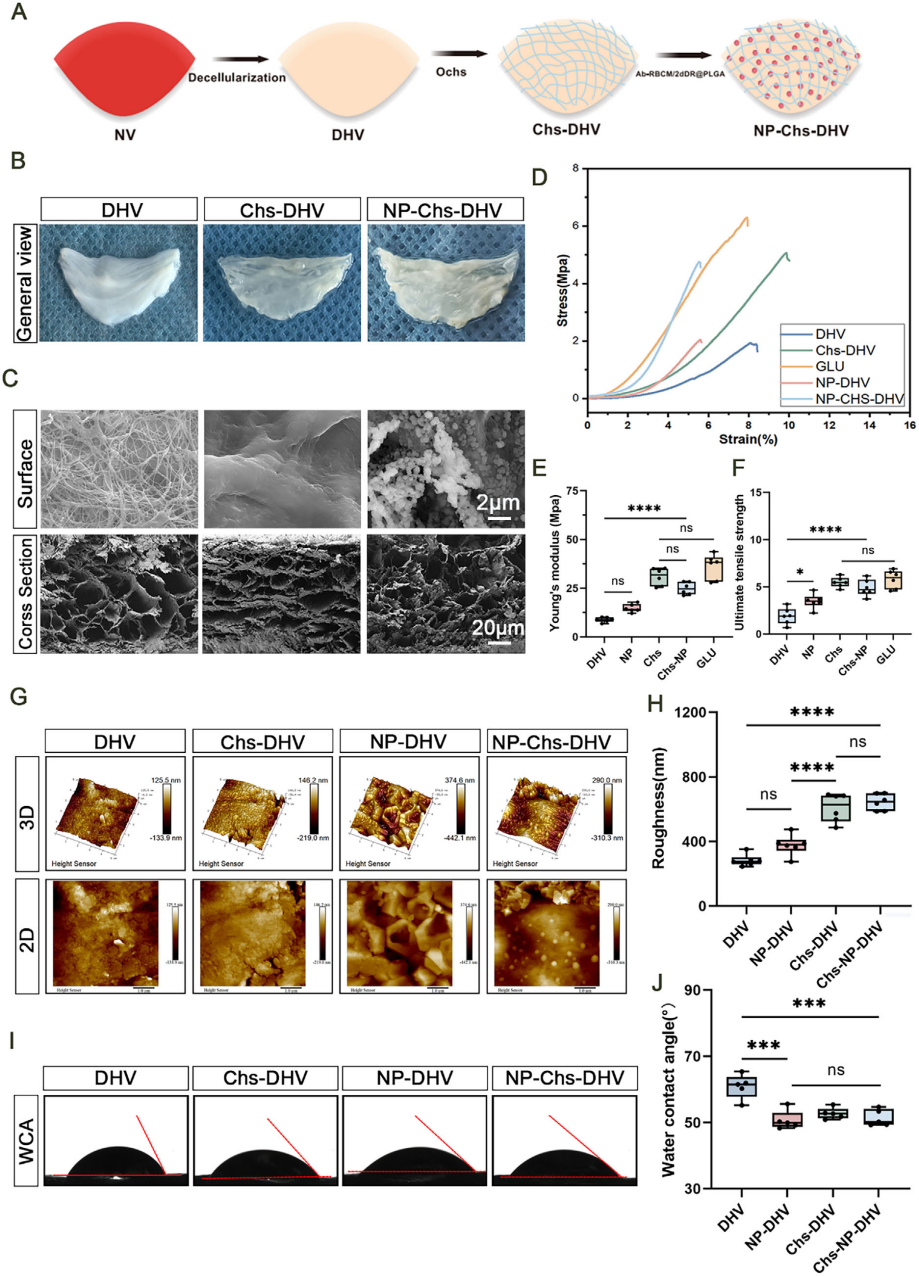
Construction and characterization of the scaffolds. A) Schematic illustration showing the steps for preparing different scaffolds. B) General view of the scaffolds. C) Topology of the scaffolds observed by scanning electron microscopy (SEM), showing that the surface fiber structure of the DHVs was flattened and that an NP structure was formed after modification. Scale bars: 2 µm (Surface), 20 µm (Cross Section). D) Strain–stress curves. E) Young's modulus. (*n* = 6). F) Ultimate tensile strength of the scaffolds. (*n* = 6). G) Atomic force microscopy images of the scaffolds. H) Surface roughness measured by atomic force microscopy. (*n* = 6). I) Images of water droplets in contact with the scaffolds. J) Water contact angle (WCA) of the scaffolds. (*n* = 6). Significance levels are denoted as ns, non‐significance; *P < 0.05; ***P < 0.001; ****P < 0.0001.

To further investigate the specific mechanisms by which Ab‐RBCM/2dDR@PLGA NPs promoted EC behavior, transcriptome sequencing was performed on ECs treated with Ab‐RBCM/2dDR@PLGA NPs and the control group. Gene Ontology (GO) and Kyoto Encyclopedia of Genes and Genomes (KEGG) pathway enrichment analyses revealed significant enrichment of regeneration‐associated pathways following treatment with 2dDR NPs, including EC development, platelet‐derived growth factor receptor β signaling, and mitogen‐activated protein kinase (MAPK) signaling pathways (Figure 2E,F). Western blot analysis was performed to validate the bioinformatic prediction of MAPK pathway activation. The phosphorylation levels of ERK, P38, and JNK were markedly increased following treatment with 2dDR NPs, whereas the application of an MAPK inhibitor reversed these effects. These results confirmed that 2Ddr NPs regulate EC behavior through the activation of the MAPK signaling pathway (Figure S2, Supporting Information). Notably, the epidermal growth factor receptor (EGFR), a common upstream regulator of these three pathways, was identified as a potential direct target of 2dDR. To identify this, we intersected the genes enriched in these three pathways and found the EGFR among the candidates. Molecular docking analysis (**Figure** **3**G) showed that the EGFR exhibited the lowest binding energy with 2dDR, which was significantly lower than that of the other candidate molecules (Figure S3, Supporting Information), suggesting that the EGFR may directly mediate the effects of 2dDR on these signaling networks. This finding reveals the molecular interaction of 2dDR with the EGFR, leading to the activation of downstream proliferation‐related pathways such as MAPK, and provides a theoretical basis for its multidimensional role in promoting endothelial repair.

To confirm that the EGFR is the direct target of 2dDR NPs, the EGFR‐specific inhibitor CP673451 was introduced into the co‐culture system, establishing four experimental groups: control (untreated), 2dDR NPs, 2dDR NPs + CP673451, and CP673451 alone. Transwell migration assays (Figure 2H,I) demonstrated that the addition of CP673451 significantly weakened the pro‐migration effect of 2dDR NPs, resulting in fewer migrating cells compared with those in the 2dDR NP group, although still higher than that in the control group. CCK‐8 proliferation assays (Figure 2J) further confirmed that CP673451 treatment markedly reduced the proliferative activity of cells in the 2dDR NP group, showing no significant difference compared with that in the CP673451 alone group. Consistently, qPCR analysis (Figure 2K) showed that CP673451 significantly inhibited the 2dDR‐induced upregulation of Ki67, VEGF, and VEGFR2 mRNA expression. Together, these results confirm that blocking the EGFR signaling pathway can partially reverse the pro‐migration and pro‐proliferation effects of 2dDR NPs, providing experimental evidence that the EGFR is a key direct target of 2dDR.

To further verify whether the EGFR mediates the biological effects of the 2dDR NPs, EGFR activation and silencing experiments were performed. Treatment with the EGFR agonist CN009543V mimicked the pro‐migratory and pro‐proliferative effects of 2dDR@RBCM‐PLGA, as demonstrated by Transwell migration, CCK‐8 proliferation, and qPCR assays, which showed increased expression of Ki67, VEGF, and VEGFR2 (Figure S4A–F, Supporting Information). Conversely, EGFR knockdown via siRNA significantly attenuated EC migration and proliferation, accompanied by the reduced expression of Ki67, VEGF, and VEGFR2 (Figure S4G–J, Supporting Information). Western blot analysis further demonstrated that treatment with 2dDR@RBCM‐PLGA and the EGFR enhanced the phosphorylation of ERK, P38, and JNK, whereas EGFR silencing suppressed these phosphorylation events (Figures S4E,K, Supporting Information). Collectively, these findings confirm that 2dDR@RBCM‐PLGA exerts its pro‐endothelial effects primarily through the activation of the EGFR–MAPK–VEGF/VEGFR2 axis.

### 2.3 Construction of the Pro‐Proliferative/Pro‐Migratory Scaffold Incorporating 2dDR NPs

Simple EDC crosslinking of NPs onto decellularized valves can induce partial self‐crosslinking; however, this approach is insufficient to significantly improve mechanical properties and long‐term stability.^[^ ^55^, ^56^ ^]^ Chondroitin sulfate (Chs), when used as a crosslinking agent, offers notable advantages for the crosslinking treatment of decellularized valves, particularly in enhancing the mechanical performance. Therefore, Chs was used to modify TEHV scaffolds. Potassium permanganate was employed to oxidize the amino groups of Chs, converting them into aldehyde groups and generating oxidized Chs. This oxidation enabled the aldehyde groups to react with the carboxyl groups on the DHV surface, resulting in the formation of Chs‐modified DHV (Chs‐DHV) complexes. Subsequently, EDC/NHS crosslinking was used to attach antibody‐functionalized erythrocyte membrane–PLGA NPs to the scaffold surface, yielding the complete NP‐Chs‐DHV system.

Figure 3A illustrates the construction of the DHV scaffold crosslinked with NPs and Chs. Figure 3B shows the general appearance of the scaffolds. Following the modification, the originally translucent DHV sheets turned white, primarily due to the color of the oxidized Chs. SEM analysis showed that the wavy fiber structure of the DHVs became flattened, and erythrocyte membrane NPs were visible on their surfaces (Figure 3C). The cross‐section of the modified scaffold retained a loose, honeycomb‐like porous structure without significant changes. Fourier‐transform infrared spectroscopy (Figure S5, Supporting Information) indicated that after crosslinking with oxidized Chs, the O─H and N─H absorption peaks at 3279 cm^−1^ shifted, suggesting changes in the hydrogen bonding between DHV molecules. Previous studies have employed the ratio of the relative intensities of amides I and II to measure protein structural differences.^[^ ^57^ ^]^ The integral results showed that the amide I/amide II ratios of the DHV and Chs samples were 1.57 and 1.86, respectively, suggesting the significant depletion of amino groups on the DHV surface and confirming successful crosslinking. The Chs‐NP sample exhibited a marked reduction in the absorption peak intensity at 3262 cm^−1^, indicating that the erythrocyte membrane NPs were successfully crosslinked to the Chs material surface via ─NH_2_, ─COOH, and other structural groups. Full XPS spectra (Figure S6, Supporting Information) for each group of valves revealed the presence of C1s, N1s, O1s, and P2p peaks across all samples. The P2p peak (≈133.58 eV) was amplified, showing that the NP‐Chs‐DHV group exhibited a higher P2p peak than the other two groups, indicating an increased phosphorus content. ICP‐MS analysis showed that simultaneous crosslinking of Chs and NPs (Chs‐NP) led to a marked decrease in the sulfur content, while the phosphorus content remained unchanged, indicating a reduced Chs conjugation efficiency and supporting the superiority of the sequential crosslinking strategy (Figure S7, Supporting Information). The release curve of 2dDR from NP‐Chs‐DHV demonstrated that the scaffold gradually released 2dDR locally after implantation, maintaining an effective concentration for promoting EC proliferation and migration for up to 15 days (Figure S8, Supporting Information), with cumulative release amounts ranging from 0.1 to 0.3 µm. Red fluorescence for CD47 and green fluorescence for the goat antibody were observed on the NP‐Chs scaffold, confirming the successful modification of the antibody‐engineered erythrocyte membrane NPs on the scaffold surface (Figure S9, Supporting Information). These results indicate that the DHV scaffold, modified with Chs and antibody‐engineered erythrocyte membrane NPs, was successfully constructed. The NP structure increased the roughness of the scaffold surface, facilitating enhanced cell adhesion and expansion, thereby providing favorable conditions for endothelialization. Its natural glycosaminoglycan properties can also interact with collagen fibers to inhibit calcification and retain bioactive components in the valve matrix that promote endothelialization, such as laminin and fibronectin. This strategy not only addresses mechanical shortcomings but also creates a composite scaffold system that integrates mechanical compatibility with repair‐promoting functionality, laying the structural foundation for the synergistic effects of 2dDR NPs.

### 2.4 Characterization of the Scaffolds

Figure 3D displays the stress–strain curve recorded during the uniaxial tensile test, with the glutaraldehyde‐crosslinked (GLUT) scaffold used as a control. The slope of the curve represents the Young's modulus of the scaffold (Figure 3E). The Young's modulus of the DHVs (8.78 ± 1.22 MPa) did not change significantly after crosslinking with NPs and remained insufficient for in vivo applications. When Chs and NPs were simultaneously crosslinked, the Young's modulus of the NP‐Chs‐DHV scaffold (24.85 ± 3.17 MPa) was significantly higher than that of the DHV scaffold and approached that of the GLUT scaffold (36.09 ± 6.42 MPa). The ultimate tensile strengths of the scaffolds exhibited a similar trend (Figure 3F). The ultimate tensile strength of the NP‐Chs‐DHV scaffold (4.88 ± 0.89 MPa) was comparable to that of the GLUT scaffold (5.79 ± 0.97 MPa) and considerably higher than that of the DHV scaffold (1.90 ± 0.87 MPa, *p* = 0.005).

The surface morphology of the scaffolds, as observed using atomic force microscopy, is shown in Figure 3G. The roughness of the DHV and NP‐DHV scaffolds was 282.8 ± 36.76 and 377.3 ± 64.16 nm, respectively, with no significant difference between them. The roughness of the Chs‐DHV and NP‐Chs‐DHV scaffolds (608.5 ± 87.8 and 643.8 ± 50.1 nm, respectively) was significantly higher than that of the DHV scaffold (Figure 3H). The water contact angle reflects the hydrophilicity of the material. Two‐step modification rendered the scaffolds more hydrophilic (Figure 3I,J), with water contact angles of 60.87 ± 3.68°, 50.67 ± 2.89°, 52.73 ± 1.71°, and 51.41 ± 2.50° for the DHV, NP‐DHV, Chs‐DHV, and NP‐Chs‐DHV scaffolds, respectively, showing statistically significant differences.

### 2.5 In Vitro EC Biological Behaviors on the Scaffolds

**Figure 4:**
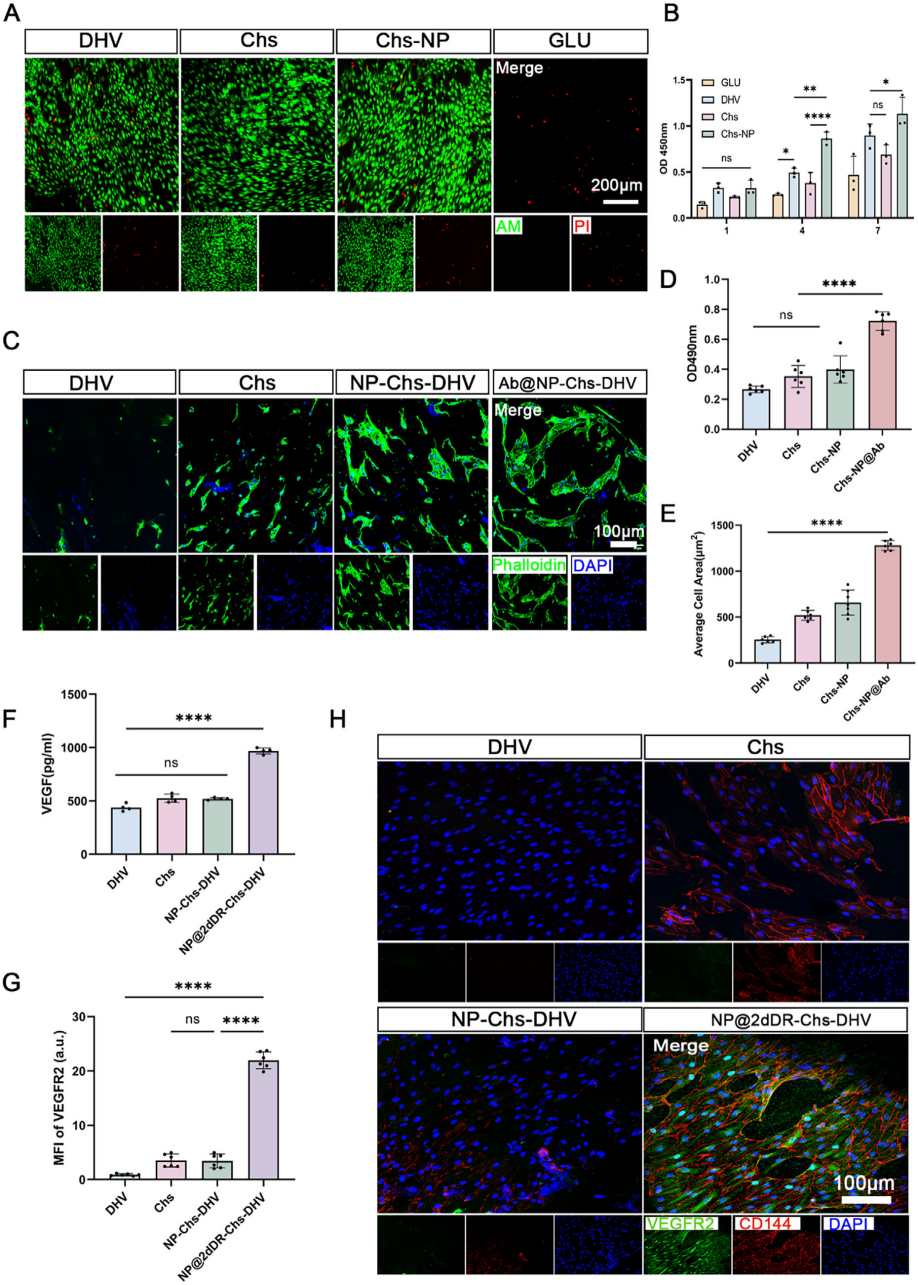
EC biological behaviors on the scaffolds. A) Live/dead cell staining of human umbilical vein ECs seeded on the scaffolds. (Scale bars: 200 µm) B) Growth curves of human umbilical vein ECs determined using the CCK‐8 assay. (*n* = 3). C) Immunofluorescence images showing endocardial cell adhesion to the scaffolds after 1 h of co‐incubation. The cytoskeleton was stained with phalloidin (green), and nuclei were counterstained with DAPI (blue). (Scale bars: 100 µm). D) Quantitative analysis of adhered endocardial cells based on OD490 absorbance measurements. (*n* = 6). E) Semi‐quantitative analysis of the cell‐spreading area. (*n* = 6). F) ELISA quantification of VEGF levels in the supernatant after 3 days of co‐culture. (*n *= 4). G) Semi‐quantitative analysis of the VEGFR2 fluorescence intensity. (*n* = 6). H) Immunofluorescence images showing the expression of CD144 (red), VEGFR2 (green), and nuclei (blue, DAPI staining) in endocardial cells. (Scale bars: 100 µm). Significance levels are denoted as ns, non‐significance; *P < 0.05; **P < 0.01; ****P < 0.0001.

The growth of human umbilical vein ECs on the DHV, Chs‐DHV, NP‐Chs‐DHV, and GLUT scaffolds is shown in **Figure** **4**A,B. On day 4, the cell count on the NP‐Chs‐DHV scaffold surpassed that on the DHV scaffold and continued to increase. PI‐AM staining revealed viable cells forming clusters on the surfaces of the DHV, Chs‐DHV, and NP‐Chs‐DHV scaffolds, indicating very low cytotoxicity. In contrast, the GLUT scaffold exhibited noticeable cytotoxicity, with almost no viable human umbilical vein ECs on its surface.

The isolated cells expressed endothelial‐specific markers (CD144, CD31, and NFATC1) and exhibited tube formation and acLDL uptake abilities, indicating the successful isolation of primary endocardial cells (Figure S10, Supporting Information). As cytoskeletal reorganization is a key determinant of cell adhesion and spreading, and the cell‐spreading area can indirectly reflect the stability of cell adhesion, we sought to verify the role of the CD144 antibody in promoting EC adhesion.^[^ ^58^, ^59^ ^]^ The scaffolds were statically incubated with ECs for 1 h, followed by thorough washing to quantify the number of adherent cells. Scaffolds modified with the CD144 antibody exhibited the highest level of endocardial cell adhesion, with statistically significant differences compared with those of the DHV, Chs‐DHV, and NP‐Chs‐DHV scaffolds lacking CD144‐antibody modification (Figure 4C,D). The extent of cell spreading was evaluated by measuring the cell area after 2 h of incubation. ECs exhibited significantly greater spreading on CD144‐modified scaffolds, with statistically significant differences relative to those on DHV, Chs‐DHV, and NP‐Chs‐DHV scaffolds without CD144‐antibody modification (Figure 4E). After culturing the scaffolds with ECs for 3 days, VEGF expression was quantified using ELISA. VEGF expression was highest in the scaffolds loaded with 2dDR NPs, showing statistically significant differences compared with those in the DHV, Chs‐DHV, and NP‐Chs‐DHV scaffolds without 2dDR loading (Figure 4F). Immunofluorescence staining revealed the highest VEGFR2 expression in ECs cultured on scaffolds loaded with 2dDR NPs, compared with those in ECs cultured on the DHV, Chs‐DHV, and NP‐Chs‐DHV scaffolds without 2dDR loading (Figure 4G,H).

### 2.6 Hemocompatibility of the Scaffolds

Regarding the in vivo performance, the interaction between the TEHV scaffold and blood triggers the coagulation cascade, leading to platelet adhesion and activation.^[^ ^60^ ^]^ The hemocompatibility and anticoagulant performance of the scaffolds were evaluated under both dynamic blood flow and static whole blood conditions.

**Figure 5:**
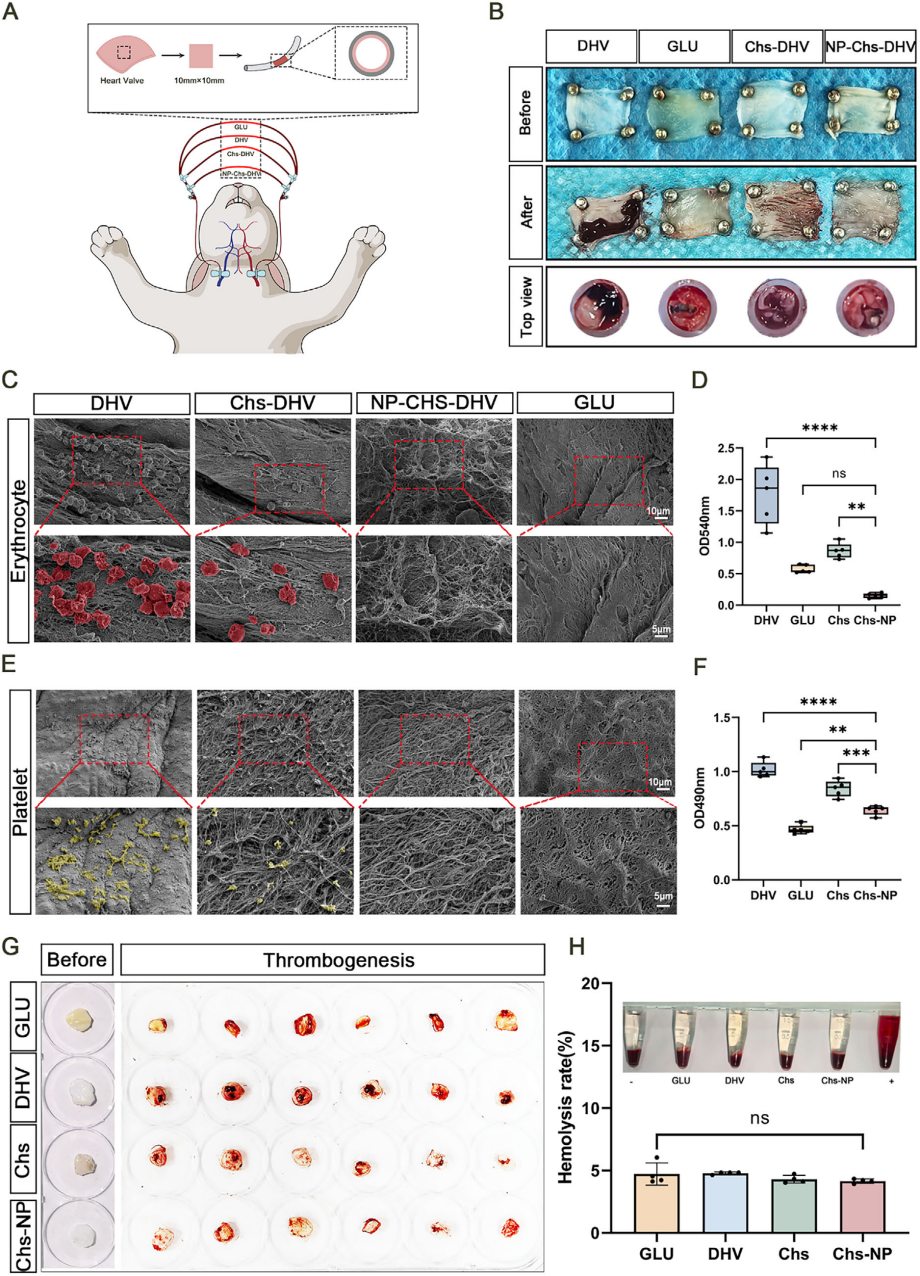
Hemocompatibility evaluation of the scaffolds. A) Schematic of the in vitro carotid–jugular bypass model. B) General view of thrombus formation on the scaffolds before and after 2 h of circulation. C) SEM image of erythrocytes on the scaffolds. Scale bars: 10 µm (upper), 5 µm (down). D) Quantification of thrombus on the scaffolds. (*n* = 5). E) Thrombogenesis of the scaffolds after incubation with whole blood. Scale bars: 10 µm (upper), 5 µm (down). F) Lactate dehydrogenase assay for the quantitative detection of platelets on the scaffolds. (*n* = 3). G) Representative SEM image of platelets on the scaffolds after in vitro incubation. H) Lactate dehydrogenase assay for the quantitative detection of platelets on the scaffolds. (*n* = 3). Significance levels are denoted as ns, non‐significance; **P < 0.01; ***P < 0.001; ****P < 0.0001.

**Figures** **5**A and S11 (Supporting Information) show a schematic of the in vitro carotid artery–jugular vein bypass model used to assess the anticoagulant performance under physiological flow. After 2 h of circulation, thrombus formation was observed on the DHV surface, whereas the Chs‐DHV, NP‐Chs‐DHV, and GLUT scaffolds exhibited markedly fewer thrombi (Figure 5B). SEM images further revealed extensive aggregation of erythrocytes on the DHV surface after circulation, whereas only a small number of RBCs adhered to the NP‐Chs‐DHV and GLUT scaffolds (Figure 5C). Quantitative analysis confirmed that the amount of thrombi on the DHVs was significantly higher than that on the Chs‐DHV, NP‐Chs‐DHV, or GLUT scaffolds (Figure 5D).

After incubation with platelet‐rich plasma, numerous activated platelets with extended pseudopodia were observed on the DHV surface, whereas only a few platelets adhered to Chs‐DHV and almost none to the NP‐Chs‐DHV or GLUT scaffolds (Figure 5E). Quantitative analysis using the lactate dehydrogenase assay further confirmed that platelet adhesion to NP‐Chs‐DHV was significantly lower compared with that on DHV and Chs‐DHV (Figure 5F).

The scaffolds were then incubated with fresh whole blood to examine thrombogenesis under static conditions. As shown in Figure 5G, substantial thrombus deposition was observed on the DHV surface, whereas the Chs‐DHV, NP‐Chs‐DHV, and GLUT scaffolds exhibited visibly less coagulation. The hemolysis test results (Figure 5H) demonstrated that all scaffolds had hemolysis rates below 5%, with no significant differences among the groups, indicating good blood compatibility and compliance with the ISO standard for blood‐contacting biomaterials.

Together, these findings demonstrate that the NP‐Chs‐DHV scaffold effectively inhibited thrombosis and platelet adhesion while maintaining excellent hemocompatibility under both dynamic and static blood conditions. The modified scaffold significantly reduced thrombosis and promoted EC coverage. The erythrocyte membrane was identified as a key factor in improving scaffold hemocompatibility.^[^ ^61^ ^]^ By mimicking the natural function of the erythrocyte membrane, it effectively mitigated blood compatibility issues and enhanced the ability of the scaffold to maintain its function under long‐term blood flow. Compared with existing anticoagulant drugs and hydrophobic surface strategies, erythrocyte membrane modification exhibited an improved early antithrombotic potential, as evidenced by reduced platelet adhesion, lower thrombus formation, and transcriptional signatures consistent with suppressed platelet activation.

**Figure 6:**
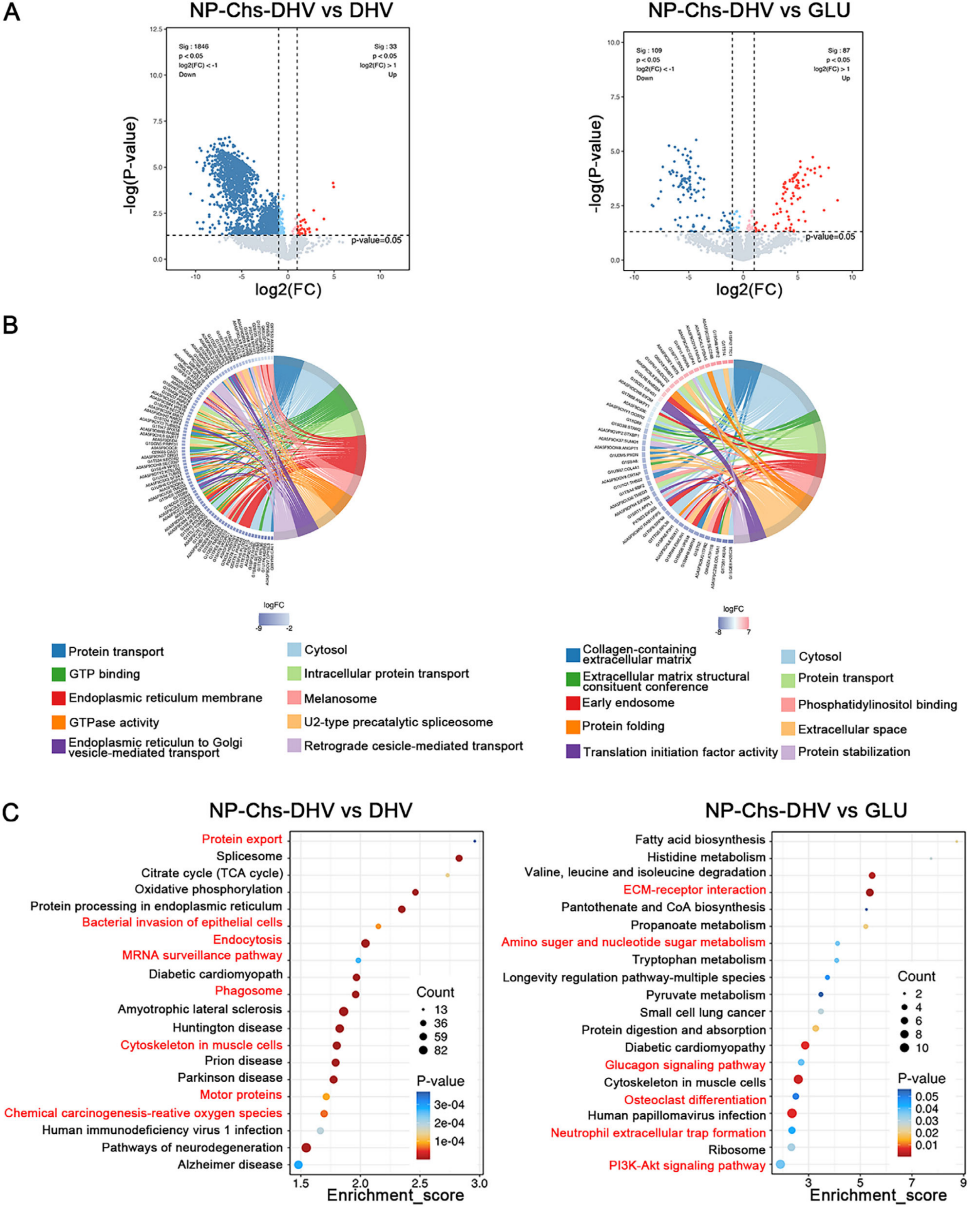
Proteomic sequencing following the carotid–jugular bypass model. A) Volcano plots displaying differentially expressed proteins (DEPs) in the three comparison groups, with down‐ and upregulated proteins indicated in blue and red, respectively, at significant thresholds of |log_2_FC| > 1 and FDR‐adjusted *p* < 0.05. B) Chord plots summarizing the GO enrichment analysis of significantly regulated proteins across the three comparison groups. C) KEGG pathway enrichment bubble plots of significantly regulated proteins in the three comparison groups.

Following the carotid–jugular bypass model, valve tissues were harvested from rabbits and subjected to proteomic sequencing to investigate the molecular alterations induced by the different treatment groups. To systematically explore the biological characteristics of the NP‐Chs‐DHV group, comparative proteomic analyses were conducted among the NP‐Chs‐DHV, DHV, and GLUT groups, integrating differential protein expression with GO and KEGG pathway enrichment (**Figure** **6**).

Volcano plots (Figure 6A) demonstrated that the NP‐Chs‐DHV group exhibited a large number of significantly downregulated proteins relative to both the DHV and GLU groups, suggesting global suppression of proteomic signatures programs associated with inflammatory activation and cellular stress. Notably, the number of downregulated proteins in the NP‐Chs‐DHV versus GLU comparison was markedly higher than that in the GLU versus DHV comparison, highlighting the superior proteomic modulation capacity of NP‐Chs‐DHV.

GO chord diagrams (Figure 6B) provided insights into the specific functional categories affected. In the NP‐Chs‐DHV versus DHV comparison, downregulated proteins were predominantly enriched in pathways such as protein transport, cytosolic ribosomes, GTP binding, and endoplasmic reticulum membrane organization, reflecting the suppression of basic metabolic and protein synthesis machinery. Functional terms including the immunoglobulin complex and adaptive immune responses were comparatively preserved, suggesting the selective maintenance of immune functions despite overall metabolic downregulation. In the NP‐Chs‐DHV versus GLU comparison, notable downregulation was observed in ECM‐related processes, such as collagen‐containing ECM organization and phosphatidylinositol binding, indicating a potential reduction in pathological extracellular remodeling and thrombosis susceptibility. In addition, the suppression of the negative regulation of post‐translational protein modification and the regulation of the production of tumor necrosis factors suggested attenuation of pro‐inflammatory signaling cascades, consistent with a broader anti‐inflammatory profile. The KEGG pathway enrichment analysis (Figure 6C) further substantiated these findings. In the KEGG enrichment analysis, several coagulation‐related pathways—including spliceosome, cytoskeleton in muscle cells, motor proteins, and endocytosis—were significantly downregulated in the NP–Chs–DHV group compared with DHV (Figure S12, Supporting Information). Mechanistically, these pathways are tightly associated with cellular activation events involved in thrombosis. The spliceosome pathway regulates RNA processing and alternative splicing of endothelial and platelet transcripts that encode coagulation‐ and inflammation‐related proteins such as tissue factor (TF) and adhesion molecules; its suppression indicates decreased protein synthesis and secretory activity, reflecting a more quiescent endothelial state.^[^ ^62^, ^63^ ^]^ Likewise, the cytoskeleton in muscle cells and motor proteins pathways represent actin–myosin dynamics, vesicle transport, and contractile force generation—key processes for platelet spreading, aggregation, and thrombus stabilization.^[^ ^64^, ^65^ ^]^ Downregulation of these pathways suggests reduced cytoskeletal remodeling and contractility. In addition, endocytosis plays an essential role in vesicular trafficking and granule mobilization in both endothelial cells and platelets, and its suppression reflects decreased vesicle transport and degranulation activity.^[^ ^66^ ^]^ Collectively, the downregulation of these pathways indicates attenuated endothelial activation and platelet responsiveness, supporting the anti‐thrombotic potential of the NP–Chs–DHV scaffold. In parallel, the comparison between NP‐Chs‐DHV and GLU highlighted the enrichment of multiple inflammation‐associated pathways, notably ECM–receptor interaction, the PI3K‐Akt signaling pathway, and neutrophil extracellular trap formation, alongside metabolic and repair‐associated pathways such as pantothenate and CoA biosynthesis, oxidative phosphorylation, and amino and nucleotide sugar metabolism. The suppression of inflammatory activation, combined with enhanced metabolic resilience, suggests that NP‐Chs‐DHV may exert a more favorable anti‐inflammatory effect than GLU. A comparative analysis was also conducted to further elucidate the differences between the DHV and GLU groups (Figure S13, Supporting Information).

### 2.7 In Vivo Performance of the Scaffolds under Hemodynamic Environment

To comprehensively evaluate the in vivo performance of the scaffold, we established a small animal model in which the scaffold was sutured to the abdominal aorta, partially mimicking the hemodynamic environment of a heart valve. This novel model allowed for a more accurate assessment of the scaffold's hemocompatibility, endothelialization, proliferation, migration, and adhesion to adjacent ECs, particularly aortic ECs. Compared with existing small‐animal models, this model more closely reflects physiological conditions and provides more comprehensive data for evaluating the scaffold performance under blood flow.

**Figure 7:**
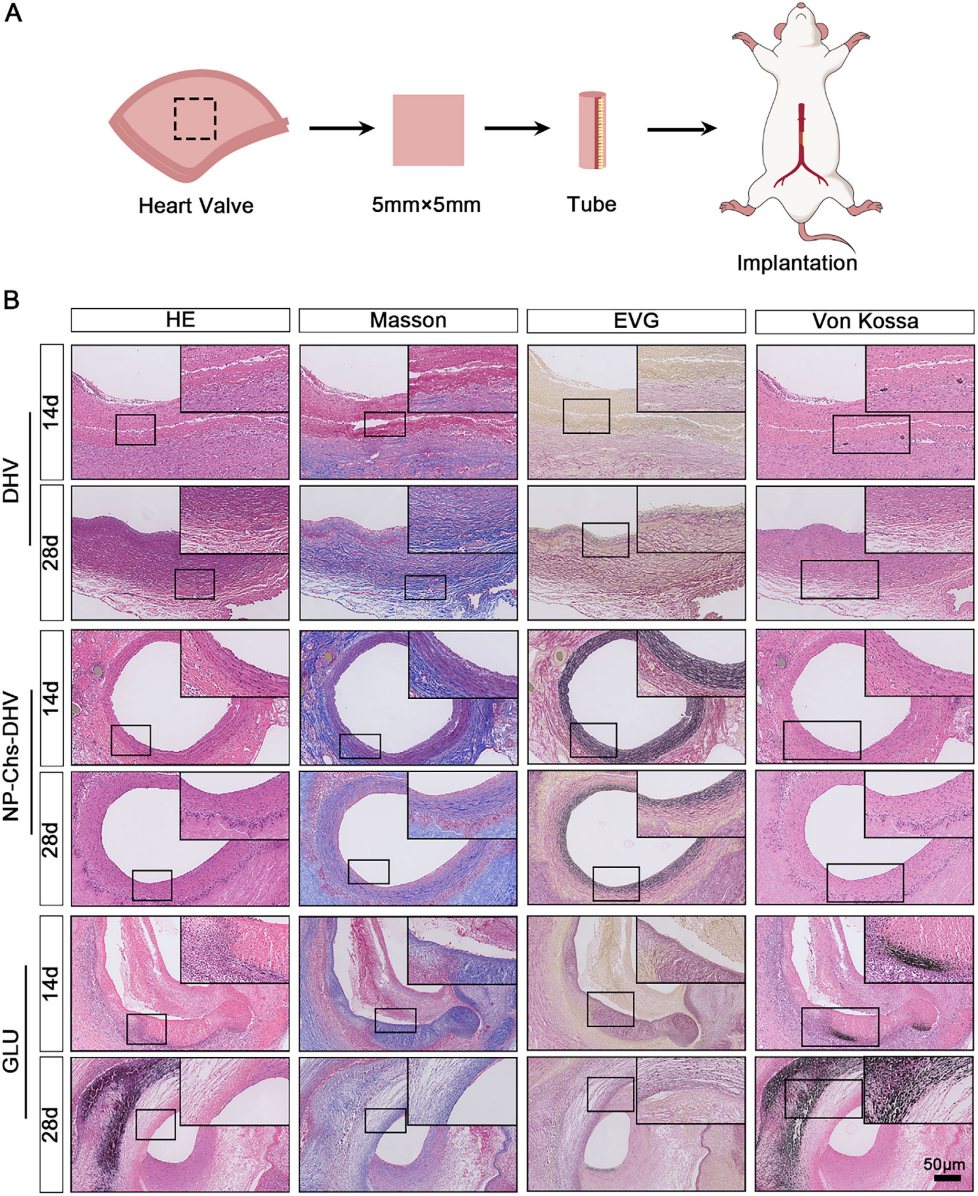
Constructive remodeling of the scaffolds after abdominal aorta implantation. A) Schematic illustration of the procedure for scaffold duct–abdominal aorta heterotopic transplantation. B) Representative histological images of the scaffolds after 7, 14, and 28 days of transplantation. H&E staining indicates cell infiltration within the scaffolds. Masson staining indicates collagen degradation and remodeling. Von Kossa staining displays calcification of the scaffolds. (Scale bars: 50 µm).

A mouse abdominal aorta transplantation model with end‐to‐end anastomosis was established to assess the performance of grafts under arterial blood flow. **Figure** **7**A illustrates the procedure for constructing the mouse model for abdominal aorta transplantation. The DHV scaffold expanded over time, forming an aneurysm after 28 days. In contrast, the other two groups showed no significant expansion, and the grafts maintained their vascular shape. Histological evaluation (Figure 7B) revealed that, compared with DHV, NP‐Chs‐DHV exhibited better collagen preservation and a smoother intima. NP‐Chs‐DHV also demonstrated significant cellular reconstruction, whereas GLU showed almost no cellular infiltration.

**Figure 8:**
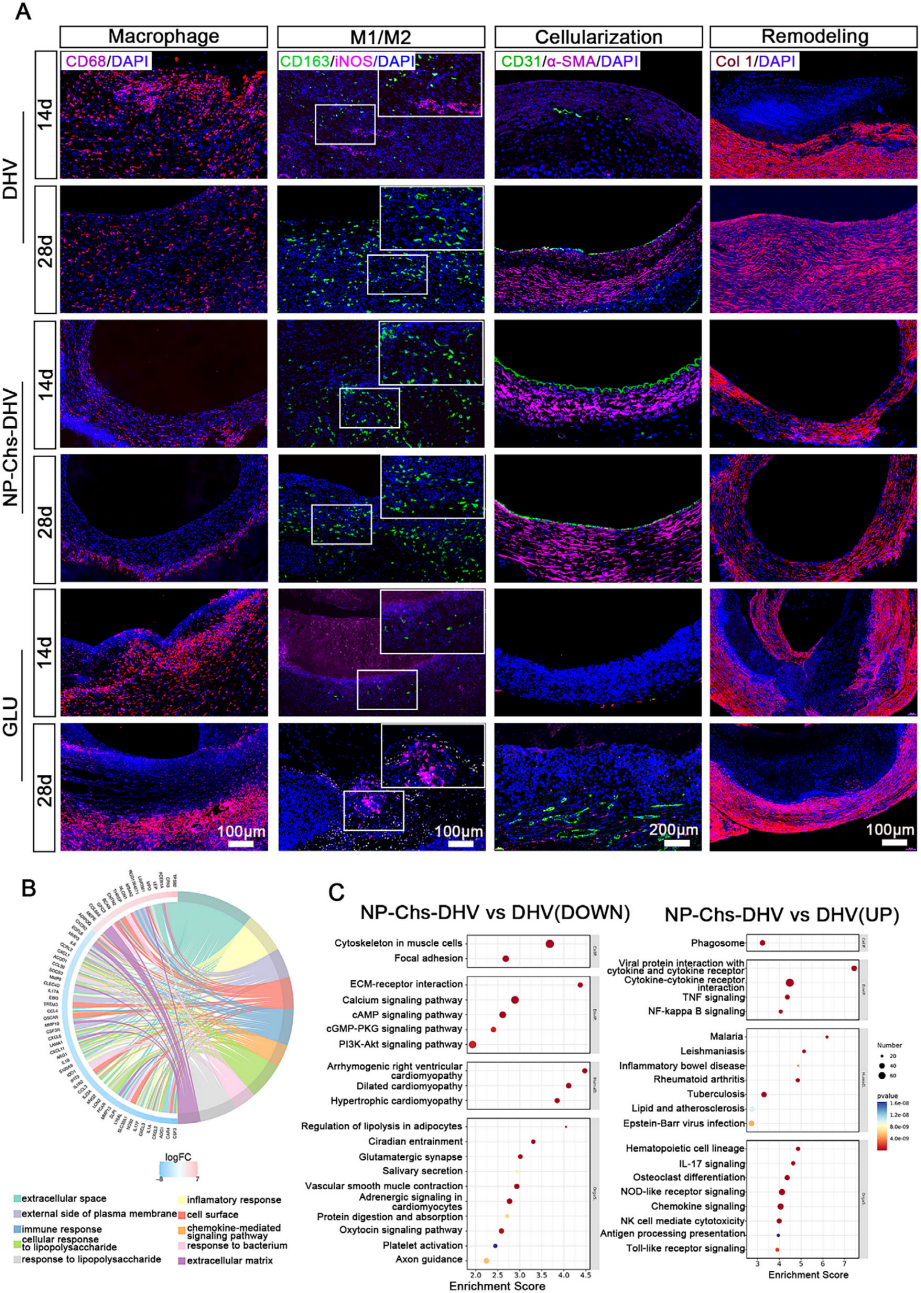
Overall performance of the scaffolds after 4 weeks of implantation. A) Immunofluorescence staining of CD68 (macrophages), iNOS (M1 subtype), and CD163 (M2 subtype) showed inflammatory infiltration of the scaffolds. Immunofluorescence staining of CD31 (ECs) and vimentin (interstitial cells) demonstrated the cellularization of the scaffolds. Collagen type I staining indicated ECM remodeling. Scale bars: 100 µm (inflammatory infiltration and remodeling), 200 µm (cellularization). B) GO chord diagram and C) KEGG pathway enrichment bubble plots for up‐ (left) and downregulated (right) genes.

The inflammation of the implanted scaffolds was visualized using CD68 (macrophages), iNOS (M1 subtype), and CD206 (M2 subtype) immunofluorescence staining (**Figure** **8**A). Overall, all scaffolds exhibited varying degrees of inflammation, with the DHV and GLUT scaffolds showing more severe inflammation than NP‐Chs‐DHV, as evidenced by increased CD68^+^ macrophage infiltration. The scaffolds mainly exhibited immunoregulatory CD206^+^ M2 macrophages, with almost no iNOS^+^ M1 macrophages after 4 weeks of implantation, indicating the resolution of acute inflammation and ongoing tissue remodeling. M1 macrophages were sporadically present in the DHV scaffolds. Compared with the DHVs, NP‐Chs‐DHV scaffolds transitioned more rapidly from acute inflammation to tissue repair and remodeling, suggesting their potential anti‐inflammatory function. In the GLUT group, most M2 macrophages were located in the surrounding dense fibrous tissue rather than infiltrating the scaffold, owing to a foreign body response caused by the non‐degradable scaffold.

The degree of endothelialization was assessed. On day 14, the NP‐Chs‐DHV scaffolds formed a confluent CD31^+^ EC monolayer with nearly complete endothelialization, whereas the DHVs showed almost no endothelialization. By week 4, the endothelialization of both the DHV and NP‐Chs‐DHV scaffolds was nearly complete, with NP‐Chs‐DHV exhibiting the highest degree of endothelialization, whereas the DHV scaffold had only a few discontinuous endothelial layers. The GLUT scaffolds showed no endothelialization at any time point. These results indicate that the NP‐Chs‐DHV scaffold achieved the fastest endothelialization rate and the highest degree of endothelialization among all groups. Mesenchymal cells also infiltrated the scaffold body, resembling the cell composition and structure of natural valves. Immunofluorescence staining revealed newly deposited collagen, indicating ECM remodeling of the scaffolds. Both DHV and NP‐Chs‐DHV scaffolds underwent degradation and remodeling, with NP‐Chs‐DHV showing the most complete remodeling compared with the DHV and GLU scaffolds. The GLUT scaffolds exhibited no cell infiltration or remodeling.

RNA‐seq analysis further confirmed the superior endothelialization potential of NP‐Chs‐DHV compared with that of unmodified DHVs. As shown in Figure 8B, the GO chord diagram revealed that NP‐Chs‐DHV was enriched in biological processes associated with cell adhesion, migration, and inflammatory regulation. Notably, key inflammatory and immune‐related pathways, including the tumor necrosis factor signaling pathway, nuclear factor‐kappa B signaling pathway, and cytokine–cytokine receptor interaction, were significantly downregulated in the NP‐Chs‐DHV group, as further validated by KEGG enrichment analysis (Figure 8C). KEGG pathway enrichment analysis of RNA‐seq data comparing the NP‐Chs‐DHV and DHV scaffolds revealed that NP‐Chs‐DHV exhibited significant upregulation of the hematopoietic cell lineage, phagosome, and osteoclast differentiation, indicating enhanced regenerative potential. Concurrently, the downregulation of cytoskeleton‐related focal adhesion, ECM–receptor interaction, and calcium/PI3K‐Akt signaling pathways suggests more stable endothelialization with reduced inflammatory and adhesion responses. Notably, the suppression of platelet activation, vascular contraction, and adrenergic signaling pathways indicates improved hemocompatibility, further confirming the early antithrombotic and anti‐inflammatory potential of NP‐Chs‐DHV compared with that of the DHVs. Concurrently, NP‐Chs‐DHV exhibited significant upregulation of pathways related to cytoskeletal remodeling and cell–matrix interactions, including the cytoskeleton in muscle cells, focal adhesion, ECM–receptor interaction, and calcium signaling pathway (Figure 8B,C). These changes were closely associated with the functionalization of the scaffold surface with CD144 (VE‐cadherin) antibodies, which specifically enhanced EC adhesion and spreading by promoting selective binding to the scaffold, thereby accelerating the early formation of the endothelial layer. Moreover, both GO and KEGG analyses (Figure 8B,C) indicated the upregulation of cellular migration‐related processes, such as cytoskeletal dynamics and calcium signaling, suggesting enhanced EC motility. This enhancement is likely driven by the controlled release of 2dDR, which facilitates EC migration and activation and promotes the formation of a continuous, functional endothelial monolayer across the scaffold surface.

## 3 Discussion

Early endothelialization, defined as the rapid coverage of the valve surface by ECs, is critical for establishing thromboresistance, reducing immune responses, and ensuring long‐term structural and functional integrity.^[^ ^67^ ^]^ It facilitates ECM stabilization and neovascularization and suppresses inflammation, thereby preventing complications such as thrombosis,^[^ ^68^ ^]^ leaflet thickening, calcification, and valve failure.^[^ ^69^, ^70^ ^]^ In this study, we developed a multifunctional nanoplatform composed of erythrocyte membrane‐camouflaged, CD144 antibody‐functionalized 2dDR@PLGA NPs (Ab‐RBCM/2dDR@PLGA), which were further crosslinked onto DHV scaffolds via oxidized‐Chs modification. This dual‐function system integrates targeted endothelial recruitment, immune evasion, pro‐migratory biochemical stimulation, and mechanical reinforcement, collectively addressing multiple barriers to rapid and stable TEHV endothelialization.

Efforts to promote the endothelialization of TEHVs have traditionally focused on recruiting EPCs or seeding autologous ECs ex vivo.^[^ ^71^ ^]^ However, these approaches have several limitations. EPCs are scarce in adult peripheral blood and require time to mature into functional ECs, and their in vivo recruitment is often inefficient and poorly controlled. Meanwhile, ex vivo cell seeding requires complex bioreactor systems, strict sterility, and is technically challenging for large‐scale or off‐the‐shelf applications. Considering these constraints, our study adopted an alternative strategy of promoting the migration of the surrounding mature ECs to the scaffold surface. By leveraging local cell dynamics rather than relying on limited progenitor pools or preseeding procedures, this approach enables rapid and functional endothelial coverage, offering a more practical and robust pathway for in situ TEHV regeneration.

To facilitate this migration‐driven endothelialization strategy, we employed 2dDR as a pro‐angiogenic and endothelial‐promoting molecule. Unlike traditional growth factors, such as VEGF, which often exhibit short half‐lives and systemic off‐target effects, 2dDR is a small endogenous sugar with low immunogenicity and good stability. Prior studies have demonstrated its ability to enhance EC migration, proliferation, and neovascularization through the activation of the EGFR–MAPK signaling pathway. In our study, the sustained release of 2dDR from the RBCM‐coated nanoplatform significantly promoted EC recruitment and surface coverage, effectively accelerating early endothelialization without the need for exogenous cell seeding or high‐dose recombinant factors. This targeted, pro‐migratory approach provides a controllable and clinically feasible solution for the in situ regeneration of TEHVs. Compared with traditional cytokines such as stromal cell‐derived factor‐1α, monocyte chemoattractant protein‐1, VEGF, and hypoxia‐inducible factor‐α,^[^ ^72^, ^73^, ^74^ ^]^ 2dDR exhibits superior stability under in vitro culture conditions, enabling the sustained stimulation of endothelial proliferation and migration.^[^ ^30^ ^]^ Notably, 2dDR is characterized by a relatively low synthetic cost, favoring its scalability for large‐scale production and applications.^[^ ^31^ ^]^ Furthermore, 2dDR has been shown to upregulate VEGF expression within ECs, establishing a positive feedback loop that promotes angiogenesis without genetic modification.^[^ ^24^ ^]^

Previous studies have shown that 2dDR promotes VEGF‐dependent angiogenesis through the activation of NADPH oxidase 2 and the nuclear factor‐kappa B signaling pathway.^[^ ^75^ ^]^ Notably, as a metabolic product of thymidine phosphorylase, 2dDR promotes the accumulation of intracellular reactive oxygen species, thereby activating stress‐response pathways such as hypoxia‐inducible factor‐1 alpha, ultimately leading to the upregulation of VEGF expression.^[^ ^29^ ^]^ In contrast, our study revealed a novel mechanism whereby 2dDR activates the EGFR, subsequently promoting endothelial repair and angiogenesis via the downstream MAPK and PDGFRB signaling pathways. These findings suggest that the EGFR may function as an upstream receptor for 2dDR, and that EGFR‐mediated signaling could indirectly enhance the VEGF/VEGFR2 activity through MAPK‐dependent crosstalk. To date, the involvement of the EGFR in 2dDR‐mediated angiogenesis has not been explored.^[^ ^76^ ^]^

In addition to its biochemical advantages, our nanoplatform leverages RBCM camouflage for both immune evasion and hemocompatibility. Unlike conventional anti‐phagocytic strategies such as PEGylation or ligand masking.^[^ ^77^, ^78^ ^]^ RBCMs provide a naturally derived membrane rich in CD47, DAF, and C8bp, which suppresses macrophage clearance and complement activation, thereby prolonging NP circulation and supporting sustained therapeutic delivery. Moreover, RBCMs significantly reduce nonspecific protein adsorption, platelet adhesion, and thrombus formation, consistent with its well‐documented bioinert interface.^[^ ^79^, ^80^ ^]^ Its native glycocalyx contributes to these effects by reducing nonspecific protein adsorption, platelet adhesion, and thrombus formation. In our study, RBCM‐coated scaffolds exhibited reduced hemolysis, prolonged coagulation times, and minimal platelet aggregation under both static and flow conditions, consistent with previous reports on the anti‐biofouling properties of RBCMs.^[^ ^81^ ^]^ Compared with synthetic coatings, such as hirudin or natriuretic peptide conjugates,^[^ ^82^, ^83^, ^84^ ^]^ which often require complex fabrication, the RBCM platform offers a simple, biocompatible, and multifunctional interface. Furthermore, this strategy allows the personalized fabrication of heart valve scaffolds using patient‐derived RBCs, potentially reducing immunogenicity and improving clinical outcomes.

Most RBCM‐based delivery systems reported to date rely on passive targeting mechanisms, such as the enhanced permeability and retention effect.^[^ ^71^ ^]^ Although some efforts have been made to improve tissue penetration using peptides, such as iRGD^[^ ^85^ ^]^ these strategies typically lack cellular specificity and are limited by the heterogeneity of the pathological vasculature. In contrast, our study employed an active targeting strategy by modifying the RBCM surface with anti‐CD144 antibodies, which specifically recognize VE‐cadherin, a cell–cell junction protein highly expressed on ECs. This targeted modification enables precise recognition and binding to resident ECs, significantly improving the cellular uptake efficiency and localization fidelity. Compared with unmodified RBCM NPs, the CD144‐functionalized system exhibited markedly enhanced uptake by ECs while minimizing off‐target internalization by macrophages. This cell‐specific targeting capability is particularly advantageous in regenerative applications, where directed migration and selective adhesion to endothelial layers are essential for promoting rapid and stable endothelialization. Thus, the integration of anti‐CD144 antibodies into RBCMs not only retains the immune‐evasive and hemocompatible properties of the native membrane but also confers an additional level of functional specificity for vascular tissue engineering.

To further validate the in vivo hemodynamic relevance of our findings, we employed a rat abdominal aorta implantation model, which is one of the most widely accepted small‐animal models for the preclinical evaluation of cardiovascular and valvular biomaterials. This model provides a physiologically relevant environment by exposing the scaffold to continuous blood flow, pulsatile pressure, and shear stress, enabling a comprehensive assessment of the mechanical stability, hemocompatibility, endothelialization, and tissue remodeling. Large‐animal orthotopic implantation is the gold standard for functional verification; accordingly, our next step will be to conduct large‐animal implantation studies to evaluate long‐term biostability, hemodynamic performance, and remodeling under physiological conditions.^[^ ^86^, ^87^, ^88^, ^89^ ^]^

Although existing studies suggest that 2dDR does not cause the pathological activation of Valvular Interstitial Cells (VICs), its potential to induce excessive proliferation or pathological epithelial–mesenchymal transition warrants further investigation. Importantly, no adverse effects were observed in the short‐term in vivo studies, and long‐term evaluations in large‐animal models are essential to assess biodegradation, remodeling, and the hemodynamic performance. In addition, the use of antibody‐based targeting may raise concerns regarding immunogenicity and production costs for large‐scale applications. Future studies should focus on evaluating the valve function under pulsatile flow, assessing biostability in orthotopic positions, and exploring alternative targeting ligands or receptor‐free strategies to improve translational applicability.

## 4 Conclusions

In conclusion, a novel TEHV scaffold was constructed by modifying it with CD144 antibody‐functionalized erythrocyte membrane–PLGA NPs and controlling the release of 2Ddr. This scaffold exhibited dual functionality, improving hemocompatibility through erythrocyte membrane modification and accelerating endothelialization by promoting cardiac EC migration and proliferation via 2dDR release. The scaffold demonstrated promising in vitro and in vivo performance, showing favorable blood compatibility, rapid endothelial coverage, and no thrombosis or calcification. Furthermore, the controlled release of 2dDR not only enhanced cardiac EC regeneration but also contributed to adaptive remodeling, enabling integration of the scaffold with native tissue. These results suggest that the newly developed TEHV scaffold can overcome the limitations of current prosthetic valves and meet clinical requirements. Moreover, its regenerative and remodeling potential holds great promise for pediatric patients requiring growing valves. Future preclinical and clinical investigations are deemed necessary to assess its potential for rapid clinical translation.

## 5 Experimental Section

### Materials

Streptavidin, Poly(lactic*‐co‐*glycolic acid) (50:50), and Chemicals relevant to decellularization were obtained from Sigma Aldrich (St. Louis, MO, USA). CLS‐PEG‐Biotin was obtained from Xi'an HUATENG PHARMA. Human VE‐Cadherin Biotinylated Antibody was obtained from R&D Systems Inc. CCK‐8 Kit was obtained from Dojindo Molecular Technologies. The lactate dehydrogenase (LDH) Cytotoxic Assay Kit was purchased from Cayman Chemical Company. LIVE/DEAD Assay Kits were purchased from Life Technologies. Primary antibodies and fluorescent secondary antibodies were purchased from BD. Fetal bovine serum (FBS), phosphate buffer saline (PBS), and Dulbecco‐modified Eagle medium (DMEM) were purchased from Gibco. EGM‐2 Endothelial Cell Growth Medium‐2 was obtained from Lonza.

### Animals

Male Sprague–Dawley (SD) rats (from Charles River Laboratory Animal Technology Co., Ltd, Beijing) were used. All these animal experiments in this study were conducted strictly according to the Guide of the Care and Use of Laboratory Animals, approved with the Laboratory Animal Ethics Committee of Tongji Medical College, Huazhong University of Science and Technology (IACUC number: 3288).

### Antibodies

Streptavidin‐PE (SA‐PE, 12‐4317‐87) was purchased from Thermo Fisher Scientific (Waltham, MA, USA). FITC‐modified detection antibody (DA‐FITC, A‐ 11 055) was provided by Abcam (Cambridge, MA, USA). COL‐1(Mouse monoclonal, ab6308 Abcam), iNOS (Mouse monoclonal, ab210823, Abcam), CD206 (Rabbit polyclonal, 16001‐1‐AP, Proteintech), vimentin (Mouse monoclonal antibody, 60330‐1‐Ig, Proteintech), CD31 (Rabbit polyclonal, ab32457, Abcam), CD68(Rabbit monoclonal, 76 437, CST), and CD206 (Rabbit monoclonal, 91 992, CST). The nuclei were stained by DAPI.

### Preparation of Antibody‐Modified Red Blood Cell Membrane (RBCM‐Ab)

To enhance endothelial cell adhesion, red blood cell membrane vesicles (RBCM) were functionalized with CD144 antibodies using the bifunctional linker Chol‐PEG‐Biotin‐3′ for targeted coupling. Whole blood from SD rats was collected and centrifuged at 2000 rpm for 10 min (4 °C) to remove serum, and the RBC pellet was washed three times with PBS (pH 7.4). The purified RBCs were resuspended in 0.25× PBS containing EDTA‐K2 and incubated at 4 °C for 30 min. After centrifugation at 12 000 rpm for 10 min (4 °C), the RBCM pellet was collected. For biotinylation, 50 µL of CLS‐PEG‐SA (100 µm) was added to 1 mL of RBCM suspension and incubated at 18 °C for 30 min. The mixture was washed three times with PBS to remove the unbound linker, resulting in biotin‐functionalized vesicles (RBCM‐Biotin). In the antibody coupling step, 50 µg of streptavidin (SA) was mixed with 10 µg of biotinylated CD144 antibody (molar ratio 1:1) and incubated for 30 min to form the SA‐antibody complex. This complex was then mixed with 1 mL of RBCM‐Biotin and incubated at 18 °C for 30 min. Through biotin‐streptavidin interaction, the antibody was anchored to the RBCM, forming RBCM‐Ab.

### Preparation of PLGA Nanoparticles (2dDR‐PLGA‐NPs)

2‐Deoxy*‐D*‐ribose (2dDR)‐loaded PLGA nanoparticles (2dDR‐PLGA‐NPs) were prepared using a double emulsion‐solvent evaporation method. A 0.1%–0.5% NaCl solution was used to dissolve 2dDR (200 mg mL^−1^) for the inner water phase (W1). The oil phase (O) consisted of a 5% PLGA 50:50 solution in ethyl acetate with 5% ethanol added to improve compatibility. For the primary emulsion (W1/O), 75 µL of W1 was added to 375 µL of O, followed by probe sonication (50 W, ice bath, 30 s). The outer water phase (W2) was prepared with a 5% NaCl solution containing 2% Poloxamer 188. For the double emulsion (W/O/W), the primary emulsion was injected into the W2 phase, followed by sonication (same parameters) and magnetic stirring (1000 rpm, 3‐4 h) to evaporate the solvent. The mixture was then centrifuged at 10 000 rpm for 20 min (4 °C), washed three times with deionized water, and lyophilized at −80 °C for 24 h. The drug loading ratio was 1:5, with 5% NaCl used to induce phase separation and Poloxamer 188 to stabilize the system. Fluorescently labeled FITC@PLGA nanoparticles were prepared by loading 0.1 wt.% FITC.

The encapsulation efficiency (EF%) and drug loading capacity (DL%) were determined using Bial's Orcinol Assay. In this method, 2dDR reacted with Bial's reagent to form a blue‐colored compound, which was then quantified by measuring its absorbance using a UV–vis spectrophotometer. The absorbance was recorded at a specific wavelength (630 nm), and the concentration of 2dDR was determined based on the absorbance value.

(1) $$EF\%=\frac{Wmir-W\sup}{Wmir}\times 100\%$$

(2) $$DL\%=\frac{Wmir-W\sup}{Wt}\times 100\%$$

Wmir means the weight of added 2dDR, Wsup means the weight of 2dDR in the supernatant, and Wt means the total 2dDR weight.

### Encapsulation of Nanoparticles with Red Blood Cell Membranes (RBCM)

RBCM‐Ab‐coated nanoparticles were prepared using the extrusion method. First, 1 mL of RBCM‐Ab vesicles was prepared from whole blood. These vesicles were then mixed with 5 mg mL^−1^ of 2dDR@PLGA nanoparticles and sonicated for 5 min. The mixture was extruded using an Avanti mini extruder through a 200 nm polycarbonate membrane for 20 passes. The resulting RBCM‐Ab/PLGA, RBCM/PLGA, and RBCM‐Ab/FITC@PLGA nanoparticles were collected and stored at 4 °C.

### Decellularized Scaffold Preparation

Porcine aortic valves were placed in TRIS‐HCl buffer (40 mm, pH 7.8) containing 2% CHAPS and 2 mmol L^−1^ TnBP, and shaken at room temperature for 24 h to remove cells. The valves were rinsed six times with sterile water, each for 10 min. The decellularization process continued with a solution containing 2% CHAPS, 2 mmol L^−1^ TnBP, 1% ASB‐14, and 2% SB 3‐10, shaking at room temperature for another 24 h.

### Dual‐Functionalized Decellularized Valve Preparation

Oxidized chitosan (OChS) was prepared using sodium periodate oxidation. The decellularized heart valve (DHV) was then incubated with a 15% OChS solution at 37 °C for 24 h, allowing the OChS to react with the amino groups on the DHV, resulting in Chs‐DHV. The OChS‐crosslinked valve was subsequently reacted with a 0.1 m EDC/NHS solution for 3 h. BCM‐Ab/2dDR@PLGA nanoparticle solution (2 mg mL^−1^) was added and incubated for 24 h. The final product was washed three times with deionized water to obtain dual‐functional nanoparticle‐crosslinked valve scaffolds.

### Proliferation and Viability Assay of HUVECs in Complex Materials

Scaffolds were trimmed into small round pieces adapted to the well of the 96‐well plate. HUVEC (1 × 106 cells mL^−1^) were resuspended and cultured on the scaffolds in the 96‐well plate. CCK‐8 kit (Biosharp, China) was used to detect cell proliferation and draw the curve at every time point (1, 3, and 7 days). On day 3 or 7, the morphology of dead or alive cells in scaffolds was observed under a confocal microscope (Olympus, Japan) after incubation with Calcein AM/PI Cell Viability Assay Kit.

### Endocardial Cell Isolation

Endocardial cells were isolated from the left ventricular outflow tract endocardial tissues of recipient hearts obtained during cardiac transplantation. The intact endocardial tissues were digested with 2 mg mL^−1^ collagenase I under continuous agitation at 37 °C for 30 min. Subsequently, the endocardial cells were gently dislodged from the tissue surface using a sterile cotton swab and resuspended in ECM supplemented with 2% fetal bovine serum (FBS), 100 µg mL^−1^ streptomycin, and 100 U mL^−1^ penicillin. The cells were cultured at 37 °C in a humidified atmosphere with 5% CO_2_, and the medium was replaced every 48 h to monitor cell adhesion and potential contamination.

### Characterization of the Scaffolds

The surface morphology and cross‐sectional view of the scaffolds were observed by SEM (Zeiss, Germany), and XPS was obtained to show the distribution of P element. The surface elemental composition and chemical state were analyzed by XPS (Thermo Fisher, USA) at a constant power of 300 W using monochromatic Al Kα radiation (*hν* = 1486.6 eV). Peak fitting was done using Avantage software.

### CD47 AND Goat Anti‐CD144 Ab Immunofluorescence Staining

CD47 and goat anti‐CD144 Ab immobilization were detected by immunofluorescence staining. Briefly, scaffolds were fixed, embedded, and sectioned. After antigen retrieval and blocking, the scaffolds were incubated overnight at 4 °C with rabbit anti‐CD47 (1:100, Affinity, DF8364), followed by incubation with Alexa Fluor 594‐conjugated anti‐rabbit antibody (1:1000, Cell Signaling Technology, 8889) and goat secondary antibody at 37 °C for 30 min. The slices were then observed under a fluorescence microscope (Zeiss, Germany).

### Mechanical Properties of the Scaffolds

Mechanical properties were evaluated by a uniaxial tensile tester (Instron, USA). Scaffolds were cut into 20 mm × 3 mm long strips along the circumferential direction, and the width, thickness, and initial length were measured using a vernier caliper. Seven samples of each group were tested under a 100 N load with a 5 mm min^−1^ extension rate until they ruptured. The stress–strain curves were recorded, and the Young's modulus and ultimate tensile strength were calculated using the accompanying software.

### Surface Roughness and Hydrophilicity of the Scaffolds

The scaffolds were freeze‐dried, and AFM (Shimadzu, Japan) and contact angle analyzer (Kruss, USA) were used to measure surface roughness and hydrophilicity of the scaffolds, respectively.

### Cytotoxicity of the Scaffolds

Scaffolds were trimmed into small round pieces with a diameter of 6 mm, sterilized by peracetic acid (0.1% w/v) for 3 h, and washed thoroughly with PBS. The cytotoxicity of the scaffolds was measured by directly culture HUVECs with a cell density of 2.5 × 10^4^ mL^−1^ on the scaffolds in a 96‐well plate. Roswell Park Memorial Institute (RPMI) 1640 medium (Hyclone, USA) was used for HUVECs culture with 10% fetal bovine serum (Gibco, USA). Cell proliferation curve was depicted by CCK‐8 (Biosharp, China). Briefly, at every time point (0, 1, 3, 5, and 7 days), scaffolds were rinsed by PBS for three times and transfer to a new 96‐well plate. Then, scaffolds were incubated with 10% CCK‐8 working solution for 3 h, and the absorbance of supernatant was measured at 450 nm. At day 10, the Calcein AM/PI Cell Viability Assay Kit (Beyotime, China) was used, and cell morphology and viability were observed under a confocal microscope (Olympus, Japan).

### Adhesion Assay of Platelets

Platelet‐rich plasma (PRP) was collected from the blood of an SD rat, which was rested (10 min) and centrifuged (1500 rpm, 20 min). Scaffolds were incubated with PRP solution (37 °C, 1 h). For quantification, after washed with PBS, samples were incubated with 0.5% Triton‐100 for 30 min. And collected supernatant was detected by the kit of LDH assay (Beyotime BioTechnology).

### Hemolysis Rate Assay

Citrated blood of the SD rat was washed 5 times with normal saline until the supernatant was transparent. Red blood cells (RBCs) were collected after centrifuging at 4000 rpm for 10 min. Samples including scaffolds, negative, and positive controls were respectively incubated with RBC solution consisting of normal saline and RBCs at 37 °C for 1 h. The collected supernatant of each sample was measured at 540 nm by a microplate reader. The hemolysis rate was calculated as:

(3) $$\frac{\mathrm{ODsc}\mathrm{affo}\mathrm{ld}\;\mathrm{group}-\mathrm{ODne}\mathrm{gati}\mathrm{ve}\;\mathrm{control}}{\mathrm{ODpo}\mathrm{siti}\mathrm{ve}\;\mathrm{control}-\mathrm{ODne}\mathrm{gati}\mathrm{ve}\;\mathrm{control}}\times 100\%$$

### In Vivo Assessment by the Model of Abdominal Aorta Implantation

The model of rat abdominal aorta implantation was used to evaluate the performance of the scaffolds (DHV, GLU, Chs‐NP‐DHV) under the hemodynamic environment. After anesthetizing the rats, an incision was made along the linea alba of the abdominal skin. Abdominal tissue was bluntly separated, and the abdominal aorta was fully exposed and separated from a vein. The proximal and distal ends of the free abdominal aorta were carefully clamped with parallel clips, and then the abdominal aorta was cut. The 8‐0 prolene sutures were used to make an end‐to‐end anastomosis of the scaffold duct and abdominal aorta. After the anastomosis was completed, the aortic clips were opened, and the abdomen was closed layer by layer.

To minimize regional variability, all explanted valve tissues were longitudinally cut through the middle portion, and sections collected from the central region were subjected to hematoxylin–eosin (HE), Masson, and immunofluorescent staining at 14 and 28 days to evaluate extracellular matrix remodeling, histocompatibility, immune response, and endothelialization.

### Western Blot

Specimens of Endocardial cells were lysed in RIPA solution added with inhibitors of proteinase and phosphatase. BCA assay (Beyotime) was used to make quantification of protein. Extracts of proteins were separated on gels and transferred to the PVDF membrane. This membrane was washed with TBST buffer, blocked with nonfat milk, and incubated overnight with primary antibodies at 4 °C and secondary antibody for 1 h at room temperature. After being washed with TBST and incubated with the ECL kit, bands were detected with a chemiluminescence system.

### Statistics

All data were presented as mean ± standard deviation (SD). No specific data pre‐processing (e.g., transformation or outlier evaluation) was performed. The sample size (*n*) for each experiment, representing the number of independent biological replicates, was provided in the corresponding figure legends. Statistical comparisons between two groups were performed using an unpaired, two‐tailed Student's *t‐*test. For comparisons among multiple groups, one‐way or two‐way analysis of variance (ANOVA) was used, followed by Tukey's post‐hoc test for multiple comparisons. All statistical tests were two‐sided, and a *P*‐value of less than 0.05 was considered statistically significant (ns, not significant; *P < 0.05; **P < 0.01; ***P < 0.001; ****P < 0.0001). All statistical analyses were performed using GraphPad Prism software (version 9.0.0 for Windows, GraphPad Software, San Diego, California USA, www.graphpad.com).

## Conflict of Interest

The authors declare no conflict of interest.

## Author Contributions

X.Q., G.L., and W.W. contributed equally to this work. X.Q. contributed to the conceptualization, methodology, investigation, formal analysis, and writing the original draft. G.L. was involved in data analysis and investigation. Y.Z. contributed to writing the review and editing, supervision, and funding acquisition. N.D. provided resources and acquired funding. W.Q. contributed to resources, writing the review and editing, supervision, and funding acquisition. W.W., J.L., G.Y., S.W., X.H., Z.H., and Y.S. contributed to experimental support and data collection. All authors reviewed and approved the final manuscript.

## Supporting information



Supporting Information

## Data Availability

The data that support the findings of this study are available on request from the corresponding author. The data are not publicly available due to privacy or ethical restrictions.
