# Supplementary material for: Targeted 2‐Deoxy‐D‐Ribose Delivery by Biomimetic Nanoplatform Activates EGFR for Accelerated Heart Valve Endothelialization
Source: Adv Sci (Weinh). 2025 Nov 12;13(6):e14170. doi: 10.1002/advs.202514170 (PMC12866836; doi:10.1002/advs.202514170)
Supplement: Supplementary file 1 — Supporting Information [file ADVS-13-e14170-s001.docx]

**Supporting Information**
***Targeted 2-deoxy-D-ribose Delivery by Biomimetic Nanoplatform Activates EGFR for Accelerated Heart Valve Endothelialization***

*Xiang Qiu^1#^, Gaofeng Li^1#^, Wenyi Wan^1#^, Jinsheng Li^1^, Ge Yan^2^, Shijie Wang^1^, Xiuqi Hu^1^, Zongqi Han^1^, Yazheng Shan^1^, Ying Zhou^1^*, Nianguo Dong^1^*, Weihua Qiao^1^**

AUTHOR ADDRESS

^1^ Department of Cardiovascular Surgery, Union Hospital, Tongji Medical College, Huazhong University of Science and Technology, 1277 Jiefang Avenue, Wuhan 430022, China.

^2^ Department of Cardiovascular Surgery, The Central Hospital of Wuhan, Tongji Medical College, Huazhong University of Science and Technology, Wuhan, Hubei 430014, China.

*Corresponding authors:

Dr. Weihua Qiao, Department of Cardiovascular Surgery, Union Hospital, Tongji Medical College, Huazhong University of Science and Technology, Wuhan 430022, China.

Email: weihua_qiao@hust.edu.cn

Prof. Nianguo Dong, Department of Cardiovascular Surgery, Union Hospital, Tongji Medical College, Huazhong University of Science and Technology, Wuhan 430022, China.

Email: dongnianguo@hotmail.com

Dr. Ying Zhou, Department of Cardiovascular Surgery, Union Hospital, Tongji Medical College, Huazhong University of Science and Technology, Wuhan 430022, China.

Email: [zhouying_hust@hust.edu.cn](mailto:zhouying_hust@hust.edu.cn)

*^#^* Xiang Qiu, Gaofeng Li, and Wenyi Wan contributed equally to this work.


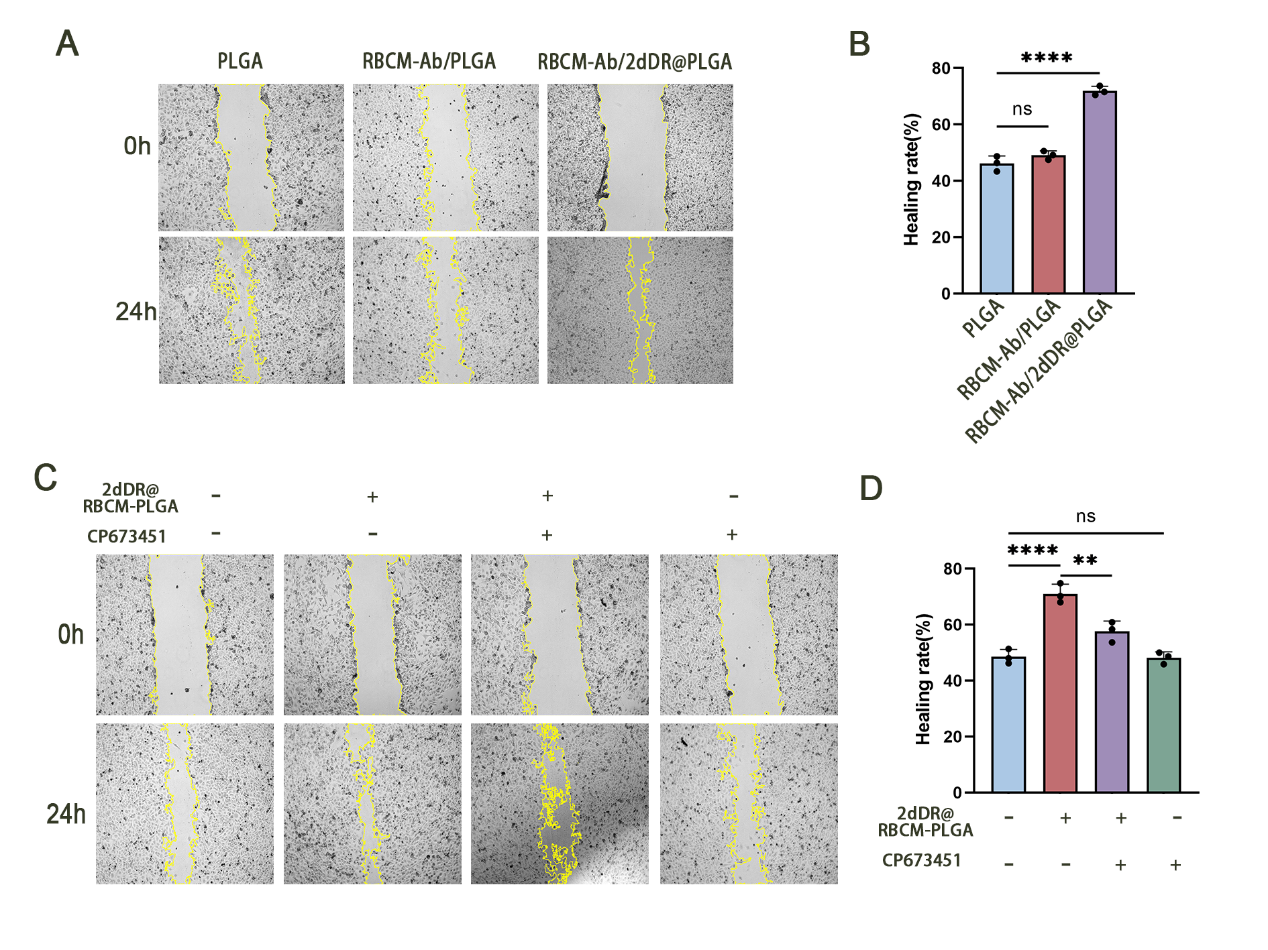


**Figure S1. Cell migration assay results.** (A) Representative scratch wound images at 0h, 12h and 24h for cells treated with PLGA, RBCM-Ab/PLGA, and RBCM-Ab/2dDR@PLGA. (B) Quantitative analysis shows that RBCM-Ab/2dDR@PLGA significantly enhances the wound healing rate. (n = 3). (C-D) Inhibition assay using CP673451 indicates that the pro-migratory effect of 2dDR@RBCM-PLGA is at least partially mediated via a relevant signaling pathway. (n = 3). Significance levels are denoted as ns, none significance; **P < 0.01; ****P < 0.0001.


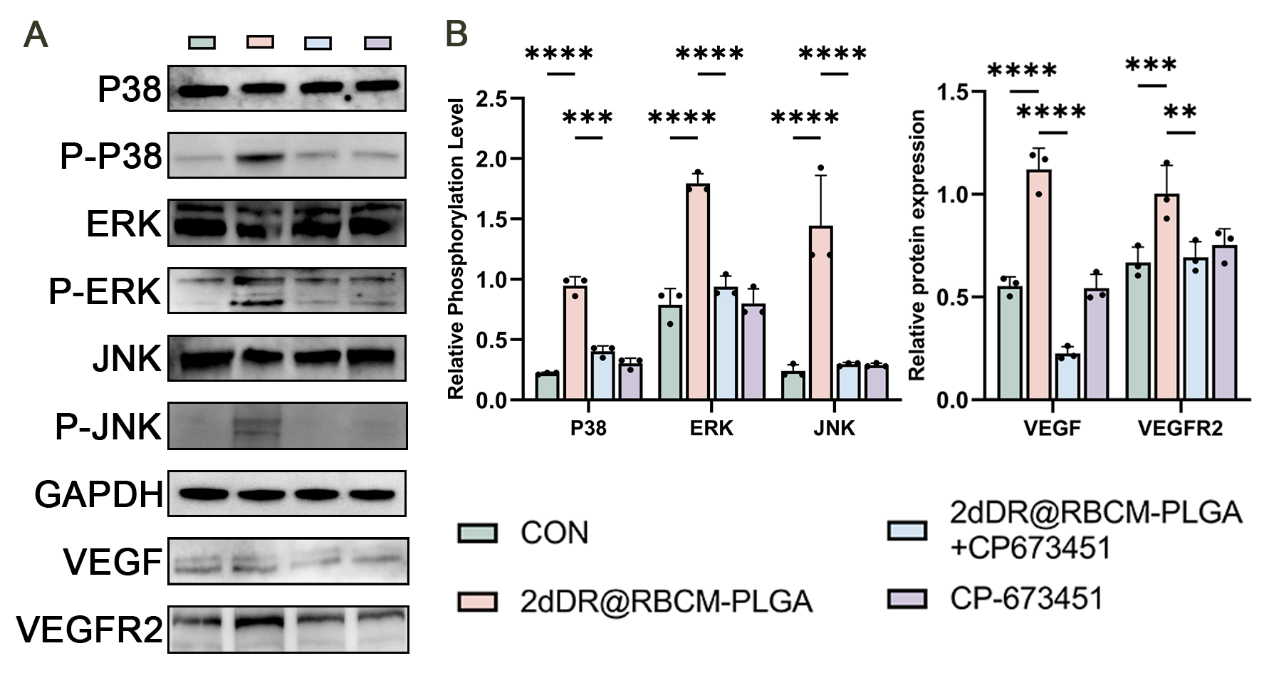


**Figure S2. Western blot validation of MAPK signaling pathway activation under different treatment conditions.** (A) and (B) Western blot analysis of P-ERK, P-P38, P-JNK, and P38 expression after treatment with control (CON), 2dDR@RBCM-PLGA, 2dDR@RBCM-PLGA+CP-673451, and CP-673451. (n = 3). Significance levels are denoted as *P < 0.05; **P < 0.01; ***P < 0.001; ****P < 0.0001.


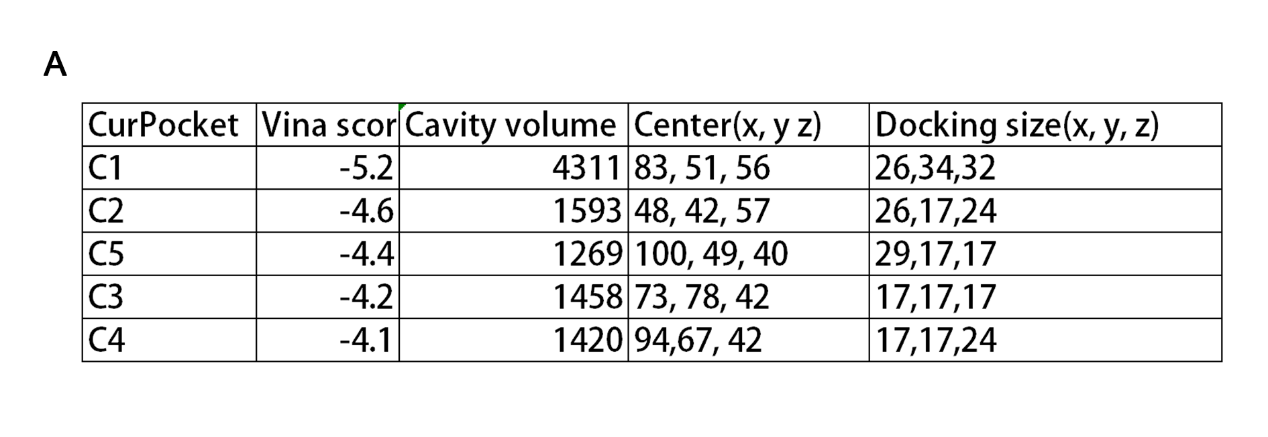


**Figure S3. Molecular docking analysis of 2dDR with EGFR.** (A)Docking results for five predicted binding pockets. Pocket C1 exhibits the lowest binding energy (–5.2 kcal/mol) and the largest cavity volume.


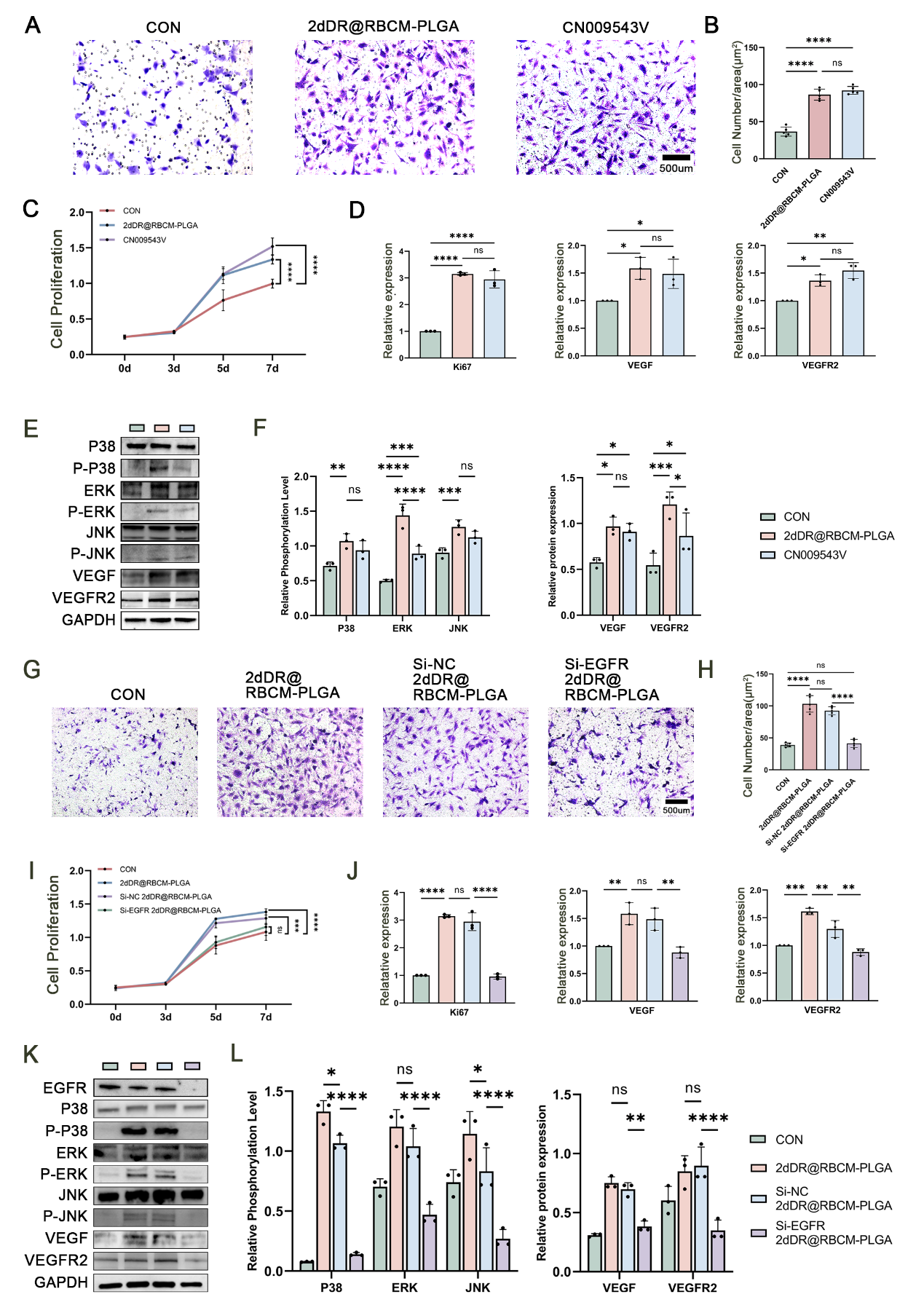


**Figure S4. EGFR mediates the endothelial activation induced by 2dDR@RBCM-PLGA nanoparticles.** (A-B) Transwell assay and quantitative analysis showing that both 2dDR@RBCM-PLGA and the EGFR agonist CN009543V enhanced endothelial cell migration compared with control. (n = 5; Scale bars: 500μm). (C) CCK-8 assay indicating increased cell proliferation in the 2dDR@RBCM-PLGA and CN009543V groups over 7 days. (n = 3). (D) qPCR analysis showing upregulated expression of Ki67, VEGF, and VEGFR2 after treatment with 2dDR@RBCM-PLGA or CN009543V. (n = 3). (E) Western blot analysis showing enhanced phosphorylation of ERK, P38, and JNK in both groups. (n = 3). (G-H) Transwell assay and quantitative results demonstrating that EGFR knockdown markedly attenuated the pro-migratory effect of 2dDR@RBCM-PLGA. (n = 5; Scale bars: 500μm). (I) CCK-8 proliferation curves showing reduced proliferation after EGFR silencing. (n = 3). (J) qPCR analysis indicating decreased Ki67, VEGF, and VEGFR2 expression following EGFR knockdown. (n = 3). (K) Western blot results showing that EGFR silencing reduced phosphorylation of ERK, P38, and JNK. (n = 3). These results collectively demonstrate that 2dDR@RBCM-PLGApromotes endothelial activation primarily through the EGFR–MAPK–VEGF/VEGFR2 signaling pathway. Significance levels are denoted as ns, none significance; *P < 0.05; **P < 0.01; ***P < 0.001; ****P < 0.0001.


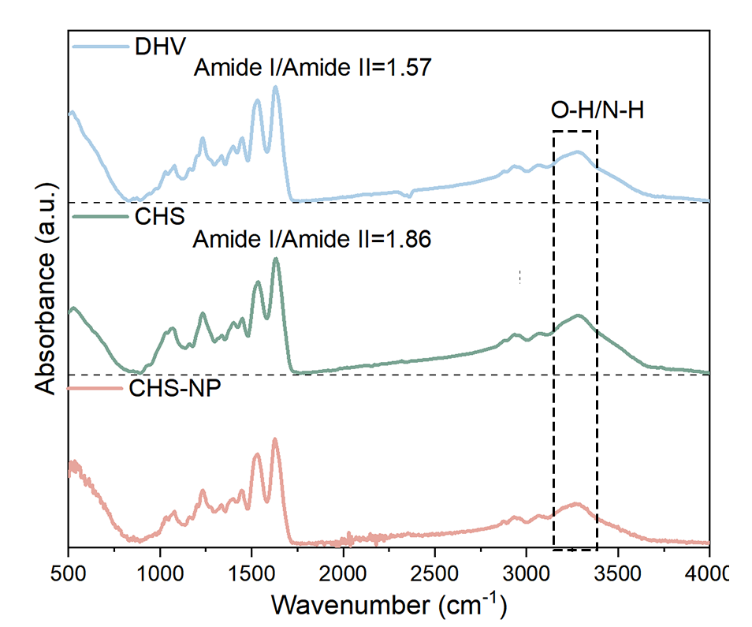


**Figure S5. FTIR spectra of DHV, CHS, and CHS-NP.** Characteristic peaks of Amide I and II and O–H/N–H stretching vibrations indicate the successful incorporation of protein and polysaccharide components. The Amide I/II ratio shifts confirm changes in chemical composition among samples.


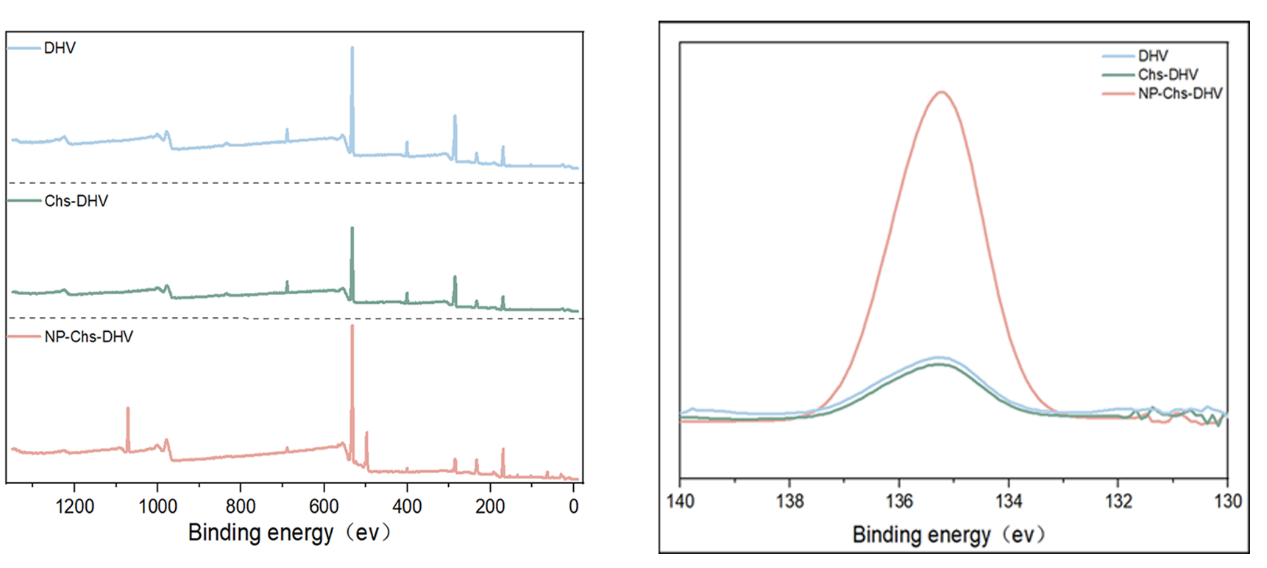


**Figure S6. XPS analysis of surface composition.** Survey spectra (left) and high-resolution P2p spectra (right) of DHV, Chs-DHV, and NP-Chs-DHV. The enhanced phosphorus signals in NP-Chs-DHV suggest surface enrichment of nitrogen- and phosphorus-containing functional groups or proteins, supporting the successful functionalization of the RBC membrane-coated nanoparticles.


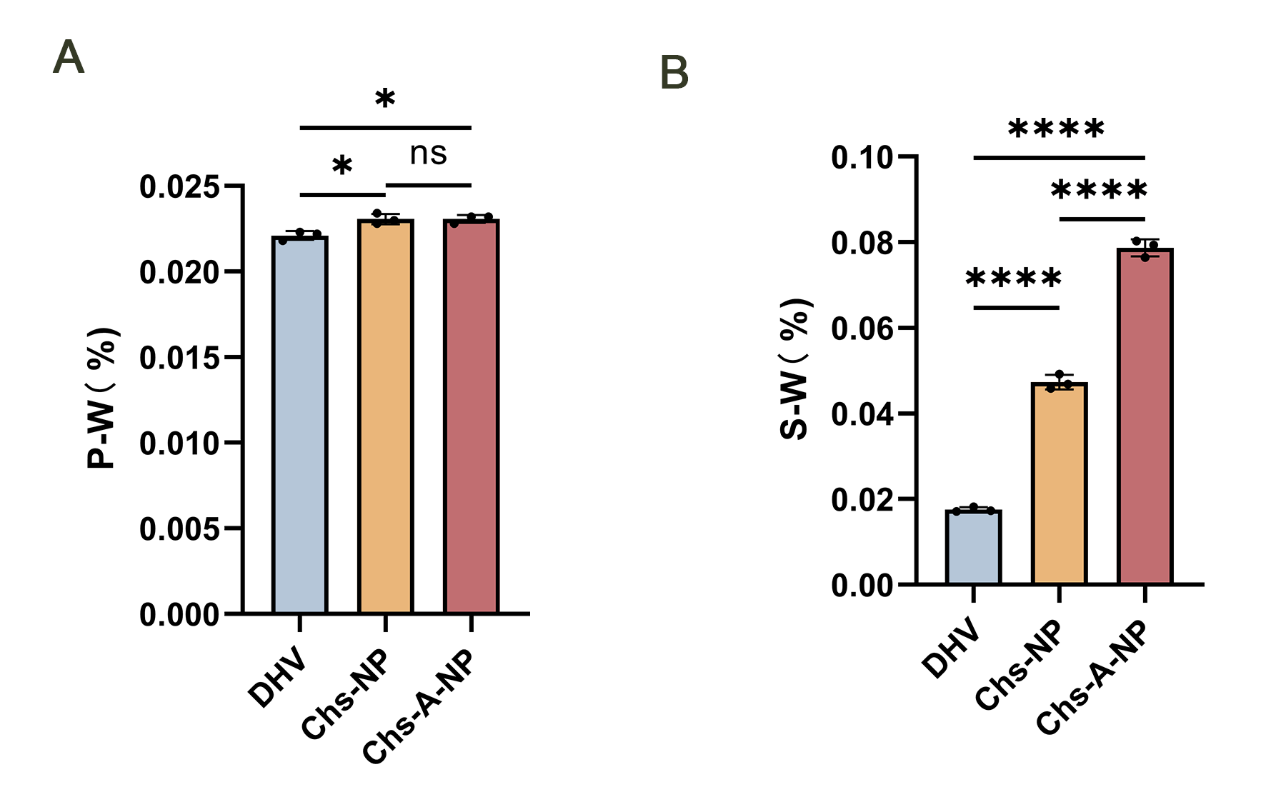


**Figure S7. Quantitative analysis of phosphorus (P) and sulfur (S) element content in different crosslinking strategies by ICP-MS.** (A) Phosphorus-to-weight ratio (P/W, %) representing the degree of nanoparticle (NP) crosslinking on DHV. (B) Sulfur-to-weight ratio (S/W, %) representing the degree of chondroitin sulfate (Chs) conjugation on DHV. (n = 3). Significance levels are denoted as ns, none significance; *P < 0.05; ****P < 0.0001.

(Note: Chs-NP represents DHV samples crosslinked with Chs and NPs simultaneously, while Chs-A-NP represents DHV samples subjected to sequential crosslinking, with Chs conjugated prior to NP attachment.)


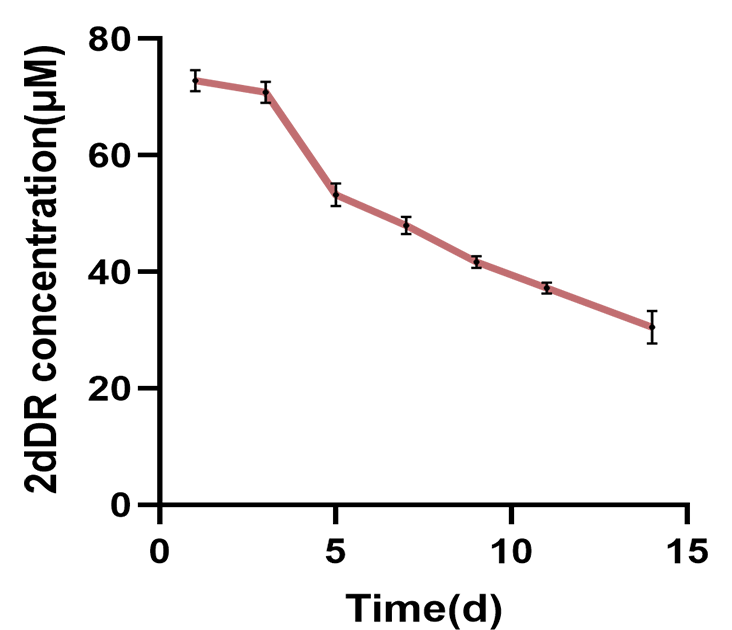


**Figure S8. In vitro release profile of 2dDR from NP-Chs-DHV.** 2dDR concentration was monitored over 14 days, showing a burst release within the first 5 days followed by a sustained slow release phase. (n = 3).


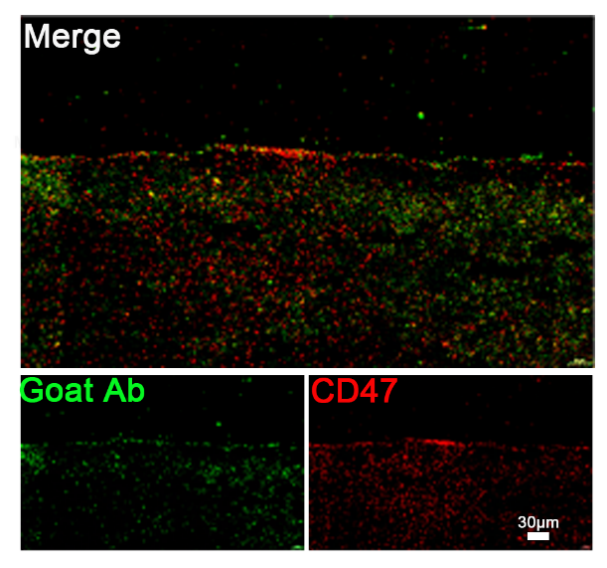


**Figure S9. Immunofluorescence verification of CD47 expression on nanoparticle surface.** Colocalization of goat anti-CD47 antibody (green) and CD47 (red) on the particle surface confirms successful nanoparticle surface modification.


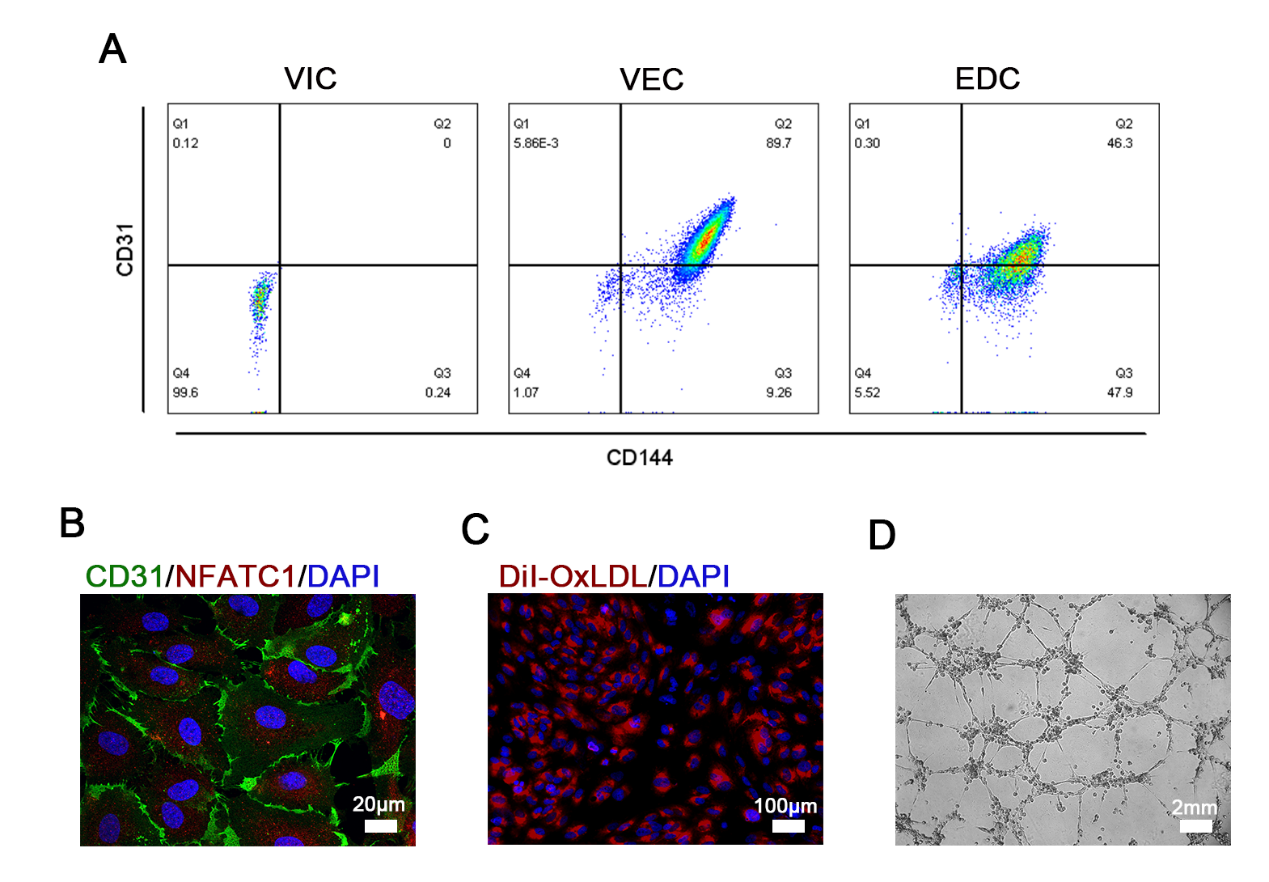


**Figure S10. Characterization of endocardial-derived cells.** (A) Flow cytometry analysis of valvular interstitial cells (VICs), valvular endothelial cells (VECs), and endocardial cells (EDCs) based on CD31 and CD144 expression profiles. (B) Immunostaining confirms endothelial marker CD31 and transcription factor NFATC1. (C) Dil-OxLDL uptake and (D) tube formation assays validate endothelial functionality. Scale bars: 20μm (B), 100μm (C), and 20μm (D).


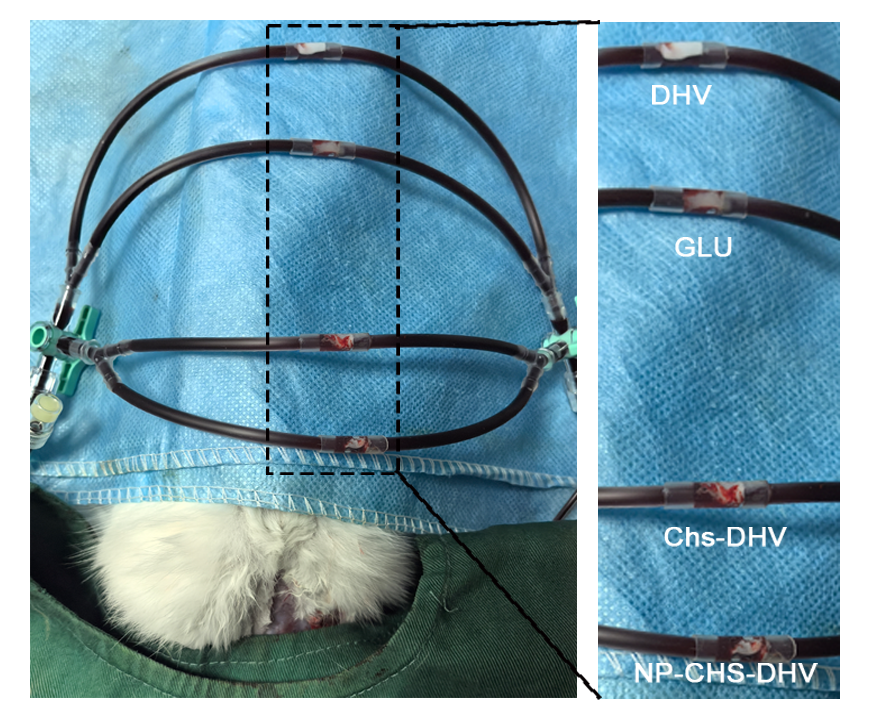


**Figure S11. Ex vivo blood loop model for thrombogenicity testing.** Different grafts (DHV, GLU, Chs-DHV, NP-Chs-DHV) were inserted into rabbit extracorporeal circuits. Visual comparison after blood flow reveals differences in clot formation and blood-material interaction.


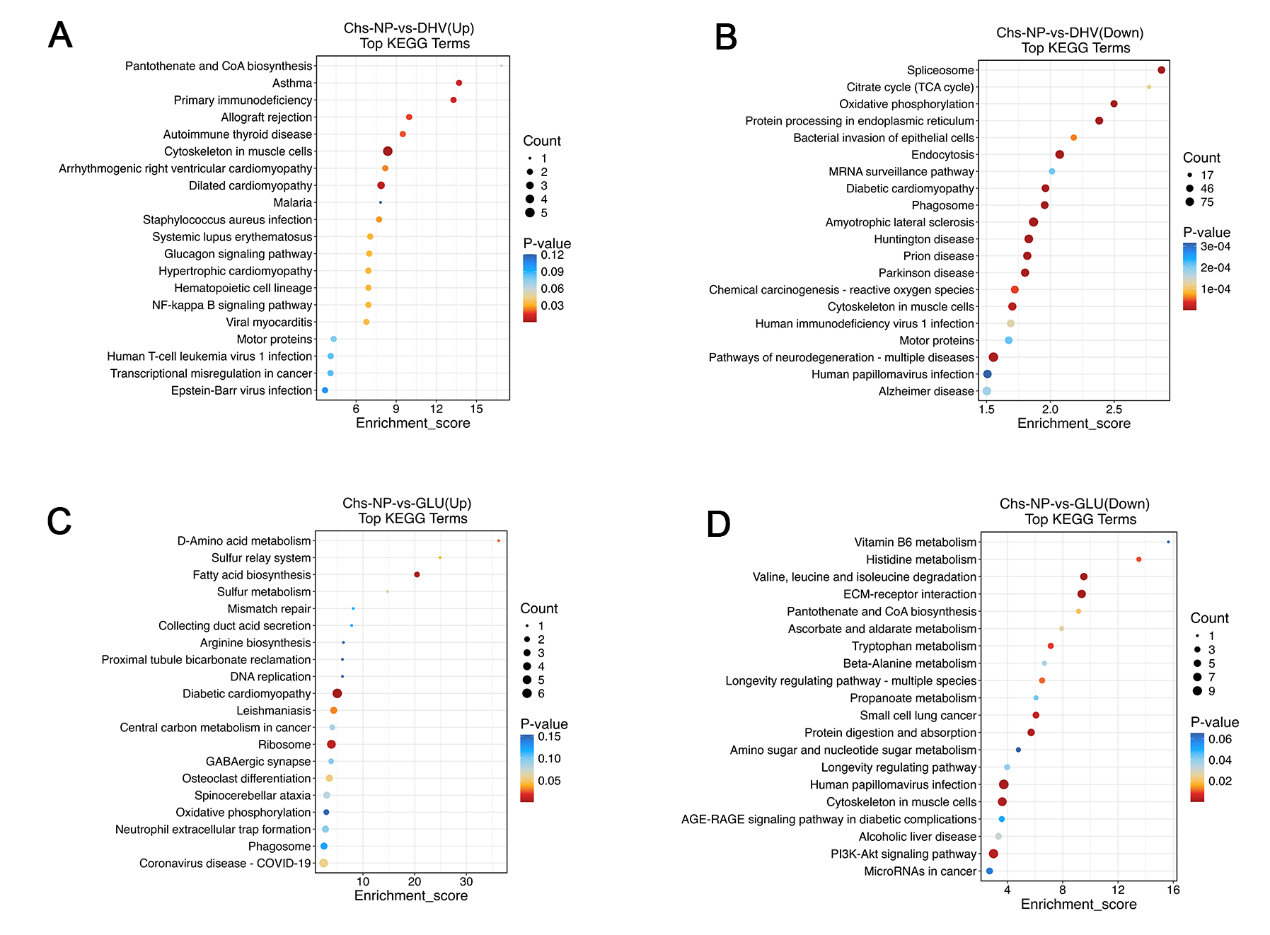


**Figure S12. KEGG pathway enrichment in Chs-NP vs DHV/GLU comparisons.** Top upregulated and downregulated KEGG pathways in Chs-NP vs DHV (A-B) and Chs-NP vs GLU (C-D) groups are shown.


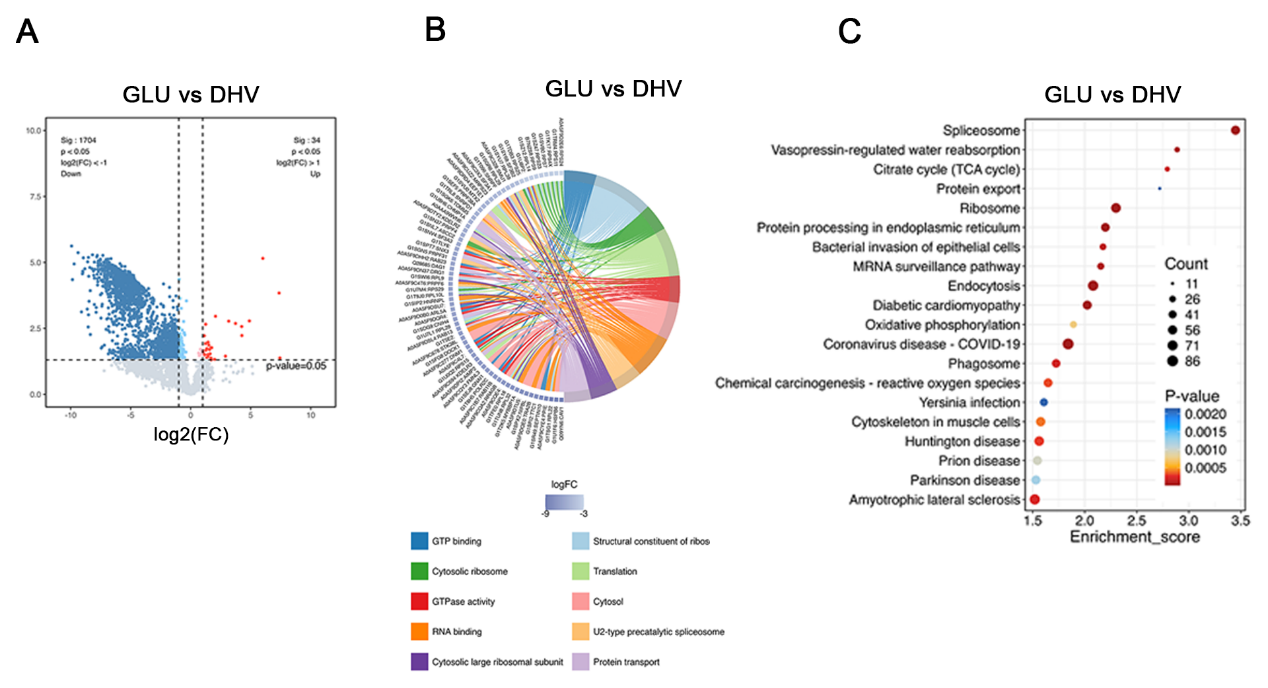


**Figure S13. Proteomic profiling of DHV vs GLU groups.** (Left) Volcano plot shows differentially expressed proteins. (Middle) GO enrichment chord plot reveals functional categories. (Right) KEGG pathway analysis highlights enrichment in metabolic and viral infection-related pathways.

| Gene | Primer Sequence (5′–3′) |
| --- | --- |
| MKI67-F | TTACAAGACTCGGTCCCTG |
| MKI67-R | TATTTGCTGTTCTGCCTCA |
| VEGF-F | ATGAACTTCACCACTTCGT |
| VEGF-R | CTGCTCTACCTCCACCAT |
| VEGFR2-F | TTACTTCTGGTTCTTCTAACG |
| VEGFR2-R | AGCAGGAATCAGTCAGTAT |
| GAPDH-F | GGTGGAATCATATTGGAACAT |
| GAPDH-R | GAGTCAACGGATTTGGTC |

**Table S1. List of primers used for quantitative real-time PCR (qRT-PCR).** Forward (F) and reverse (R) primers were designed for MKI67, VEGF, VEGFR2, and GAPDH genes. All primer sequences are written in the 5′ to 3′ direction.
